# An all-round AI-Chemist with a scientific mind

**DOI:** 10.1093/nsr/nwac190

**Published:** 2022-09-08

**Authors:** Qing Zhu, Fei Zhang, Yan Huang, Hengyu Xiao, LuYuan Zhao, XuChun Zhang, Tao Song, XinSheng Tang, Xiang Li, Guo He, BaoChen Chong, JunYi Zhou, YiHan Zhang, Baicheng Zhang, JiaQi Cao, Man Luo, Song Wang, GuiLin Ye, WanJun Zhang, Xin Chen, Shuang Cong, Donglai Zhou, Huirong Li, Jialei Li, Gang Zou, WeiWei Shang, Jun Jiang, Yi Luo

**Affiliations:** Hefei National Research Center for Physical Sciences at the Microscale, School of Chemistry and Materials Science, University of Science and Technology of China, Hefei 230026, China; School of Information Science and Technology, University of Science and Technology of China, Hefei 230026, China; Hefei National Research Center for Physical Sciences at the Microscale, School of Chemistry and Materials Science, University of Science and Technology of China, Hefei 230026, China; Hefei National Research Center for Physical Sciences at the Microscale, School of Chemistry and Materials Science, University of Science and Technology of China, Hefei 230026, China; Hefei National Research Center for Physical Sciences at the Microscale, School of Chemistry and Materials Science, University of Science and Technology of China, Hefei 230026, China; School of Information Science and Technology, University of Science and Technology of China, Hefei 230026, China; School of Information Science and Technology, University of Science and Technology of China, Hefei 230026, China; School of Information Science and Technology, University of Science and Technology of China, Hefei 230026, China; School of Information Science and Technology, University of Science and Technology of China, Hefei 230026, China; School of Information Science and Technology, University of Science and Technology of China, Hefei 230026, China; School of Information Science and Technology, University of Science and Technology of China, Hefei 230026, China; School of Information Science and Technology, University of Science and Technology of China, Hefei 230026, China; School of Information Science and Technology, University of Science and Technology of China, Hefei 230026, China; Hefei National Research Center for Physical Sciences at the Microscale, School of Chemistry and Materials Science, University of Science and Technology of China, Hefei 230026, China; Hefei National Research Center for Physical Sciences at the Microscale, School of Chemistry and Materials Science, University of Science and Technology of China, Hefei 230026, China; Hefei National Research Center for Physical Sciences at the Microscale, School of Chemistry and Materials Science, University of Science and Technology of China, Hefei 230026, China; Hefei National Research Center for Physical Sciences at the Microscale, School of Chemistry and Materials Science, University of Science and Technology of China, Hefei 230026, China; Hefei JiShu Quantum Technology Co. Ltd, Hefei 230026, China; Hefei JiShu Quantum Technology Co. Ltd, Hefei 230026, China; Hefei JiShu Quantum Technology Co. Ltd, Hefei 230026, China; School of Information Science and Technology, University of Science and Technology of China, Hefei 230026, China; Hefei National Research Center for Physical Sciences at the Microscale, School of Chemistry and Materials Science, University of Science and Technology of China, Hefei 230026, China; Hefei National Research Center for Physical Sciences at the Microscale, School of Chemistry and Materials Science, University of Science and Technology of China, Hefei 230026, China; Hefei National Research Center for Physical Sciences at the Microscale, School of Chemistry and Materials Science, University of Science and Technology of China, Hefei 230026, China; Hefei National Research Center for Physical Sciences at the Microscale, School of Chemistry and Materials Science, University of Science and Technology of China, Hefei 230026, China; School of Information Science and Technology, University of Science and Technology of China, Hefei 230026, China; Hefei National Research Center for Physical Sciences at the Microscale, School of Chemistry and Materials Science, University of Science and Technology of China, Hefei 230026, China; Hefei National Laboratory, University of Science and Technology of China, Hefei 230088, China; Hefei National Research Center for Physical Sciences at the Microscale, School of Chemistry and Materials Science, University of Science and Technology of China, Hefei 230026, China; Hefei National Laboratory, University of Science and Technology of China, Hefei 230088, China

**Keywords:** AI-Chemist, computational brain, mobile robot, machine reading, all-round research

## Abstract

The realization of automated chemical experiments by robots unveiled the prelude to an artificial intelligence (AI) laboratory. Several AI-based systems or robots with specific chemical skills have been demonstrated, but conducting all-round scientific research remains challenging. Here, we present an all-round AI-Chemist equipped with scientific data intelligence that is capable of performing basic tasks generally required in chemical research. Based on a service platform, the AI-Chemist is able to automatically read the literatures from a cloud database and propose experimental plans accordingly. It can control a mobile robot in-house or online to automatically execute the complete experimental process on 14 workstations, including synthesis, characterization and performance tests. The experimental data can be simultaneously analysed by the computational brain of the AI-Chemist through machine learning and Bayesian optimization, allowing a new hypothesis for the next iteration to be proposed. The competence of the AI-Chemist has been scrutinized by three different chemical tasks. In the future, the more advanced all-round AI-Chemists equipped with scientific data intelligence may cause changes to the landscape of the chemical laboratory.

## Teaser

An all-round AI-chemist with scientific mind has been created, which is able to automatically perform all research tasks and self-controlled by its computational brain.

## INTRODUCTION

Empowering a robot with a scientific mind to help humans decipher the high-dimensional correlations of the complex world, reduce the Try–Error development cost of new materials and even realize interstellar exploration and colonization has been a fantasy in many science fiction stories [1,2]. The recent advancement in automated chemical experiments by robots has offered a glimpse of hope [3–15]. An automated chemical machine system (called Chemputer) has been developed to integrate literature reading, protocol customization, organic synthesis and characterization [9,10], while another more generalized system could carry on Lego-like automated organic synthesis [11,12]. In 2020, a cloud-native chemical platform with artificial intelligence (AI) as its controller was reported of performing organic retrosynthesis based on previously available data [13,14]. Meanwhile, the research group led by Cooper has pushed a step further to develop a mobile robotic chemist with the abilities to perform experiments faster than humans and to select photocatalysts with Bayesian optimization [15]. But, they also specifically mentioned in their article that the robotic search employed in their work does not capture existing chemical knowledge, nor include theory or physical models. A better approach as they suggested is to equip it with a computational brain and to fuse theory and physical models with autonomous searches [15]. Indeed, only after inserting a computational brain can the robot truly be self-controlled by its scientific mind to conduct all-round scientific research.

**Figure 1.**
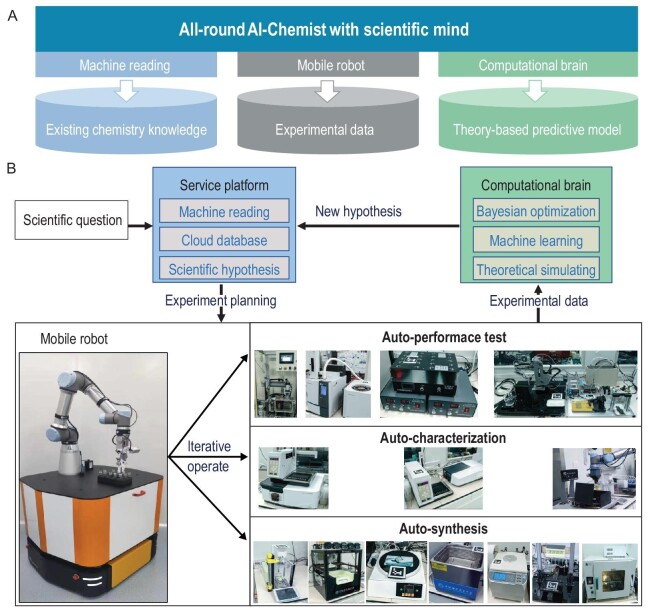
Design of the all-round AI-Chemist with a scientific mind. (A) Three modules of the AI-Chemist: a machine-reading module, a mobile robot module and a computational brain module. (B) The workflow of the AI-Chemist and the functions of each module.

Along this line, we have built an all-round AI-chemistry laboratory that can (i) propose scientific hypothesis and generate experiment plans using existing knowledge, (ii) execute the complete experimental procedure (synthesis, characterization and performance testing) for multiple chemical tasks and (iii) build predictive models exploiting theoretical computations and experimental data feedback, so as to execute all-round chemical research with intelligence. Figure 1A outlines the overall architecture of the all-round AI-Chemist. This AI-Chemist with human-like research wisdom consists of three modules, including a machine-reading module to capture existing chemical knowledge by automatically reading massive amounts of chemical literature, a mobile robot module to produce experimental data by executing various chemical experiments and a computational brain module to generate physics/theory-based predictive models by carrying out theoretical calculations. The completion of the three modules leads to the birth of an all-round AI-Chemist with a scientific mind.

## RESULTS AND DISCUSSION

The workflow of this all-round AI-Chemist forms a complete closed loop (Fig. 1B) and is controlled by home-developed system software (Supplementary Fig. S1). When the AI-Chemist is prompted with a scientific question raised by a human researcher, its service platform with a user-friendly graphical user interface (GUI) would propose a scientific hypothesis after capturing existing knowledge by machine reading of massive amounts of literature. This service platform possesses the ability of accessing cloud databases and designing experiment plans and has a web-based feature that enables it to easily manage tasks and remotely monitor the mobile robot. The experiment plans generated by this service platform are provided to the mobile robot and various smart chemical workstations. Control system software based on a Robot Operating System (ROS) [16] is developed to control the mobile robot with a six-degree-of-freedom robotic arm to move around and operate all apparatuses. The laboratory hosting the robot and apparatus is divided into an auto-synthesis area, an auto-characterization area and an auto-performance-testing area, respectively. All instruments were designed to operate using vials of the same type so as to realize the combination of multiple apparatuses to form a complete workstation. Many previous synthesis robots have mostly optimized experimental plans according to previous data in the literature [9] or the data generated by the robot itself [15], while the AI-Chemist can ‘think’ more deeply. Thanks to the computational brain it possesses, it can not only capture existing chemical knowledge and accept experimental feedback, but also perform theoretical simulations, train machine-learning models and carry out Bayesian optimizations to provide global optimal solution. This robot can propose a new hypothesis and new experiment plan by itself, as well as carry out the next round of chemical experiments by itself.

**Figure 2.**
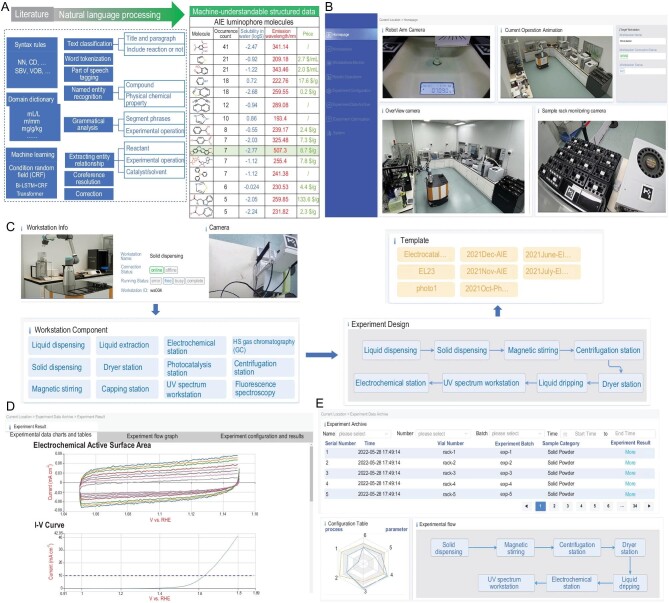
The service platform with web browser-based HMI. (A) Machine-reading module for capturing existing knowledge and transferring to machine-understandable structured data by NLP. (B) User-friendly GUI to monitor the status of chemical workstations and robots in real time. (C) Experimental workflow diagram: from scientific hypotheses to workstation status, then to experimental design and template. (D) Experimental data such as electrochemical active surface area and current–voltage curve collected and displayed on the service platform. (E) Experimental archives for building the experimental database and training the prediction model.

The first task of an AI-Chemist is to read through a large amount of literature to gain the wisdom of human chemists. By digitizing and standardizing experimental protocols written in natural language from the literature, the existing knowledge can be transferred to the robot to enrich its brain. As shown in Fig. 2A, based on syntax rules, domain dictionaries and machine learning, we build a scientific text data-mining system with eight natural language processing (NLP) steps. This system can decompose titles, paragraphs and sentences by text classification, locate important regions by word tokenization and part-of-speech tagging, name entity recognition, perform grammatical analysis, extract entity relationships and realize co-reference resolutions and error corrections. The NLP steps convert scientific texts into structured data understandable for AI. This data-mining system belongs to a service platform with a web browser-based human machine interface (HMI), which can further propose hypothesis and provide intelligent recommendation of experiment plans according to above structured data. A user-friendly GUI helps researchers to remotely monitor the status of the robot and workstations (Fig. 2B and Supplementary Fig. S2) and customize the experimental procedure by clicking and dragging on the GUI. As shown in Fig. 2C, according to the scientific hypothesis, the service platform communicates with all workstations to check their status and customizes experiment workflow. We have also generated a cloud-based chemical database to facilitate choosing chemical samples from a solid or liquid dispensing menu with a dragging-down list (Supplementary Fig. S3A). The database can also be accessed through a web interface when searching for any compound, such as terephthalic acid as shown in Supplementary Fig. S3B. Once the experiment workflows are validated, they are stored as experimental templates to a cloud database for subsequent experiment recall. The service platform can also carry out preliminary analysis of experimental data collected, and the results of characterization and performance testing are displayed and visualized as graphs whenever possible on the browser-based interface (Fig. 2D and Supplementary Figs S4–S9). These experimental data are also used to establish and update a database for subsequent prediction models and Bayesian optimizations (Fig. 2E). The AI-Chemist is able to automatically iterate experimental conditions until experimental results reach threshold parameters set previously (Supplementary Fig. S10).

**Figure 3.**
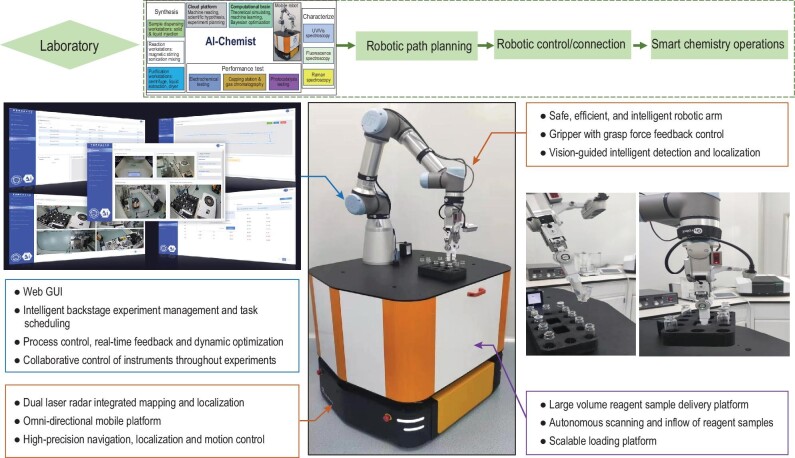
Intelligent chemical laboratory and mobile robot. The intelligent chemical laboratory with capabilities of robotic path planning, robotic control and connection, and smart chemical operations and the mobile robot equipped with control system, dual-lidar-based localization system, six-degree-of-freedom robotic arm and large scalable loading platform.

To execute the whole process of synthesis, characterization and test, we have set up a mobile robot and 14 workstations in the laboratory, including an auto-synthesis region, auto-characterization region and auto-performance-test region, respectively. When AI-Chemist executes a synthesis task, it uses the liquid dispensing workstation with an accuracy of 3 μL and the solid dispensing workstation with an accuracy of 0.1 mg to prepare the reagents (Supplementary Figs S11 and S12). It then uses the magnetic stirring workstation and sonication mixing workstation with time-control accuracy of a millisecond to make the desired reactions (Supplementary Figs S13 and S14). Finally, the purification of the products is conducted at the dryer workstation, centrifugation workstation and liquid extraction workstation (Supplementary Figs S15–S17). After completing the synthesis process, the sample vessel with products is transferred to the auto-characterization region and auto-performance-test region. Currently, the laboratory is equipped with auto-controlled UV–Vis, fluorescence and Raman spectroscopy workstations to characterize the component and structure of products (Supplementary Figs S18–S20), as well as an auto-controlled electrochemical workstation, capping workstation, photocatalysis workstation and HS gas chromatography (GC) to test the catalytic performance of products (Supplementary Figs S21–S24). Home-developed system software capable of robotic path planning, robotic control and connection, and smart chemical operations is in charge of coordinating the real-time interactions between the robot and the workstation. The mobile robot simply executes all orders in sequence and each workstation only needs to ensure an experimental operation is executed using accurate parameters and the experimental data are fed back to the system correctly. Together, the robot and the workstations form a holistic smart laboratory for the AI-Chemist (Fig. 3).

To ensure precise and interactive chemical operations, the mobile robot is equipped with a dual-lidar-based integrated mapping and localization system to obtain its localization information and laboratory map for navigation and autonomous obstacle avoidance (Fig. 3 and Supplementary Fig. S25). An omni-directional mobile platform with a global localization precision of 10 mm and max loading of 200 kg ensures the motion accuracy and load ability of the robot. A large and scalable loading and delivery platform is ready for high-throughput and multi-tasking experiments. The chemical operations are performed using a six-degree-of-freedom robotic arm (Fig. 3). It is equipped with a gripper with grasp force feedback control, depth cameras and laser sensors, and a vision-guided intelligent detection and localization system. To fulfill the requirements of high localization accuracy of the gripper in experimental operations, an independent ArUco label is attached to each experimental workstation (Supplementary Figs S26–S30). The robot can recognize the current workstation by visually identifying the label ID in the field of view and make the accurate measurement of the workstation pose according to the relative pose between the ArUco label and the target to be operated on at the experimental workstation. To handle abnormal situations during experimental operation, such as detecting and locating transparent vials in unstructured scenarios, a set of algorithms based on deep learning has been developed (Supplementary Figs S31 and S32). A task management module is of central importance in the whole robot control system. It is responsible for detecting the current status of the robot and workstations and planning experimental operations (Supplementary Figs S33 and S34). A video presents an overview of this intelligent, full-process chemical laboratory (Supplementary Video 1). Compared with existing literature reports on automated synthesis systems, characterization and related performance testing, our AI-Chemist incorporates a computational brain and chemical workstations, and is therefore able to achieve more chemistry functions and higher control precision (Supplementary Table S1).

**Figure 4.**
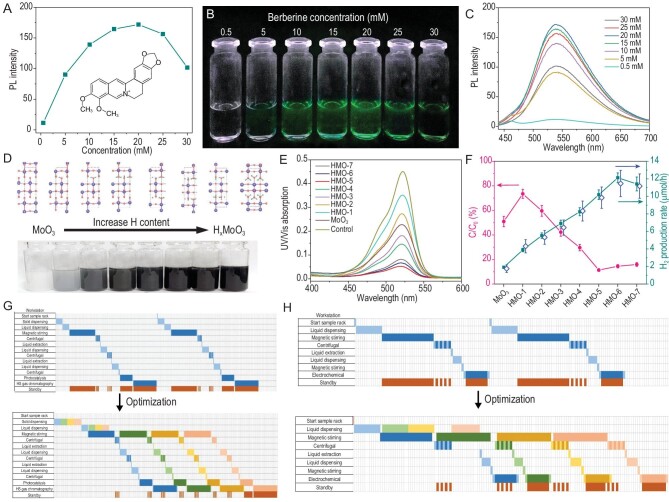
Chemical experiments performed by mobile robot and workstations. (A) Peak fluorescence intensity with increased concentrations of berberine chloride (BBR). (B) Fluorescence images of the solution in (A) under UV light (365 nm). (C) PL spectra of BBR chloride in solution with different concentrations of BBR chloride. Excitation wavelength: 405 nm. (D) Tunable hydrogenation of MoO_3_ by Cu–acid treatment with simulated crystal structure of H_x_MoO_3_ with different concentrations of H-dopants in the lattice. (E) The UV–Vis spectra of the RhB solution after photocatalytic degradation. (F) Characterization of the photocatalytic degradation efficiency of the RhB (left column, reaction conditions: [cat] = 0.1 g), solution volume: 10 mL H_2_O, [RhB] = 10^−5^ mol/L) and the yield of dye-sensitized photocatalytic H_2_ production (right column, reaction conditions: [Eosin-Y] = 0.6 mM, [TEOA] = 1.5 M, [cat] = 0.1 g, solution volume: 10 mL. All the light sources are 25 W white LED lamps). The error bars show the systematic average error and the comparison with the manual work result (blue error bar). (G) Schematic diagrams of experiment flow for dye-sensitized photocatalytic water-splitting experiment without (top) and with (bottom) the multi-task dynamic optimization. (H) Schematic diagrams of experiment flow for electrocatalytic oxygen evolution reaction experiments without (top) and with (bottom) the multi-task dynamic optimization.

Two experiments were performed to examine the hardware and software of the robot and workstations. In the first experiment, the AI-Chemist is instructed to find a biocompatible luminophore with aggregation-induced emission (AIE) characteristics [17–19]. The machine-reading module is activated: from 15 979 papers, 4865 molecules are found to relate to ‘AIE’, ‘aggregation-induced emission’ and ‘aggregation induced emission’. After data cleansing based on expertise rules (see details in ‘Methods’ section), 306 molecules are identified to be candidates as commercially available AIE luminophores. Figure 2A and Supplementary Table S2 list the top 15 molecules with high occurrence frequencies (see Supplementary Data 1 for complete list). Among them, Berberine chloride (BBR) [20,21] is the only one with an emission wavelength located in the visible region and is therefore chosen for further investigation. A series of automated experiments are conducted by the AI-Chemist to synthesis different berberine chloride solutions and measure their fluorescence. The robot weights an appropriate amount of solid berberine chloride sample using the solid dispensing workstation and then transfers it to the liquid dispensing workstation to dissolve it into a solution. The solutions’ optical properties are measured using photoluminescence (PL) spectroscopy and UV spectroscopy. As shown in Fig. 4A–C, BBR solutions of different concentrations and solvents are compared and the optimal concentration of BBR is thus identified to be 20 mM.

The second experiment is to optimize a hydrogen-doping strategy for metal oxide photocatalysts. A set of H_x_MoO_3_ samples with various amounts of hydrogenation via Cu–acid treatment [22] are produced by the synthesis workstations (Fig. 4D) and then used for photocatalytic degradation of rhodamine B (RhB) at the photocatalysis workstation. The photocatalytic reactions are monitored by the UV–Vis spectroscopy workstation (Fig. 4E): the photocatalytic degradation efficiency of RhB reaches a maximum level on the sample HMO-5. To obtain the yield of dye-sensitized photocatalytic H_2_ production during the reaction, the vials are vacuum sealed at the capping workstation and transferred to the HS GC workstation for measurement of H_2_ produced. The sample HMO-6 shows the highest H_2_ production rate, as shown in Fig. 4F. Here the average of deviation bars for all experimentally measured points are 5.5% for the H_2_ production rate and 8.3% for the RhB degradation efficiency (Supplementary Table S3), suggesting the high accuracy and repeatability of the AI-Chemist. Based on the task management module, a smart chemical system capable of multi-task dynamic optimization is developed. With its help, the total experiment time and waiting time of the robot can be significantly shortened: the total time consumption of the four sample racks of dye-sensitized photocatalytic water-splitting experiments without optimization is 1810 minutes, while the total time consumption of the four sample racks of experiments after multi-task dynamic optimization is 980 minutes (Fig. 4G and Supplementary Text).

**Figure 5.**
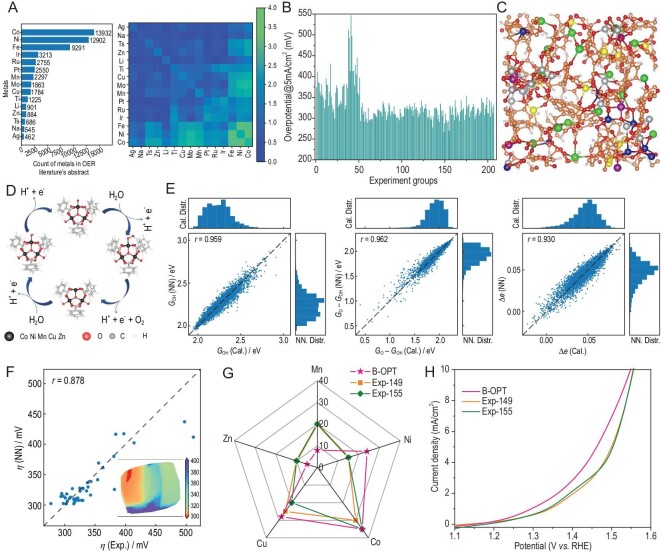
An all-round chemical research conducted by the AI-Chemist. (A) The order of metal recommendation and the frequency of metal co-occurrence by machine reading. (B) The overpotential values of 207 Try–Error experiments carried out by the mobile robot and workstations. (C) An example of the simulated structure generated by molecular dynamics. (D) The simulated OER reaction path. (E) The prediction results of three catalytic properties by neural network models, where G_OH_ is the free energy change of the hydroxyl adsorption, G_O_ is the free energy change of the oxygen atom adsorption and Δe is the charge transfer during hydroxyl adsorption. (F) The prediction results of overpotential by the neural network model calibrated by experimental data. Inset: dimensionality reduction plot by principal component analysis (PCA) for predicted overpotentials of all exhaustive samples. (G) Kiviat diagram of composition ratios and (H) polarization curves of the optimal sample suggested by the Bayesian model and the best samples by Try–Error experiments.

The above-mentioned two tasks have proven that mobile robot and smart workstations can successfully execute various experimental operations. Next, we integrate them with the service platform and computational brain to act as an AI-Chemist to conduct all-round chemical research. We take the non-noble metal oxygen evolution reaction (OER) electrocatalyst as an example. It has been shown that high-entropy alloy nanoparticles containing more than four different elements have high potential to be the choice for future electrocatalysts due to their structural stability, diversity of adsorption sites and remarkable catalytic activity [23–26]. Among them, the MIL-101 MOF is an ideal platform for different metal elements to synthesize high-entropy electrocatalysts [27]. However, the wide range of choices in element combinations poses grand challenges for developing high-entropy electrocatalysts. Even if we only want to determine a desirable metal combination, it could take hundreds of thousands of years to traverse the best composition ratio. This simple fact makes high-entropy alloy nanoparticles a suitable task for our all-round AI-Chemist with a scientific mind. Again, the service platform sorts out metal recommendations via the intelligent machine reading of ∼16 000 papers from the cloud database (Fig. 5A and Supplementary Data 2). Five non-precious metal elements are selected to construct 207 Try–Error experiments fully executed by the mobile robot on the smart chemical workstations. The home-developed smart chemical system successfully shortens the total time consumption of the four groups of experiments from 630 to 365 minutes through multi-task dynamic optimization (Fig. 4H and Supplementary Text). The experimental results summarized in Fig. 5B show that the No. 149 and No. 155 samples have the best electrocatalytic performance (Supplementary Figs S36 and S37). We directly carry out Bayesian optimizations based on the experimental data to determine the optimal composition ratios. Two optimized solutions are suggested (Supplementary Figs S38 and S39), whose composition ratios and performances are quite close to the best results of the above-mentioned experiments (Supplementary Fig. S40). This could imply that these are only local optimal solutions due to the blindness of Bayesian optimization based on experimental data [15]. To take advantage of the computational brain inserted into our AI-Chemist, we have extended our search based on theoretical calculations. Herein, ∼20 000 molecular dynamics simulations are performed by the computational brain module to generate a large number of structures (Fig. 5C and Supplementary Fig. S41) and a high-quality simulation data set through density functional theory calculations (see details in ‘Methods’ section and Supplementary Table S4). According to the structure around the metal node in the MIL-101, we calculate the overpotentials based on the reaction path shown in Fig. 5D and Supplementary Fig. S42. The trend of simulated overpotential values is consistent with the experiment, but there are systematic errors for the absolute value, which need to be corrected by combining simulations and experiments (Supplementary Fig. S43). Eventually, a machine-learning model for predicting key electrocatalytic properties from metal composition ratios is established by using the simulated data (Fig. 5E) and is calibrated with the Try–Error experimental data to obtain an accurate prediction model for real experimental overpotentials (Fig. 5F). This calibrated model can rapidly and massively predict the composition-ratio-based overpotentials (inset of Fig. 5F) that can be used to evaluate the electrocatalytic performance of high-entropy materials. Again, the Bayesian optimization algorithm is applied to narrow down the huge searching space (553 401 options) and find the optimal solution. From the Kiviat diagram, one can see the optimal composition ratio suggested by the Bayesian model is far from the best sample of the above-mentioned experiments (Fig. 5G), which indicates that the Bayesian optimization based on the combination of simulated and experimental data sets can go beyond the local optimal regions. The sample with the best composition ratio is then synthesized and characterized by the AI-Chemist and its overpotential is 231.7 mV at a current density of 5 mA/cm^2^, which is considerably better than the results of previous Try–Error experiments (Fig. 5H and Supplementary Fig. S44). We also notice that the composition ratio suggested by Bayesian optimization is almost the same as that suggested by grid-point scanning (Supplementary Fig. S45), proving that it is indeed a global optimal solution. This task has served as a very good example to illustrate the superior ability of the all-round AI-Chemist with a scientific mind. As presented in Supplementary Video 2, the scientific mind of the AI-Chemist comes from its computational brain, which is able to calculate the physical parameters based on physical models such as MD and DFT. The computational brain can further establish machine-learning models based on big data and perform a global search based on Bayesian optimization. From millions of possibilities, the new scientific hypothesis for the optimal solution was proposed and verified experimentally. We also manually synthesized the optimal material and found that its OER performance is basically consistent with the results made by the AI-Chemist (Supplementary Fig. S46), confirming that the experimental results of our intelligent system are highly stable, reliable, trustworthy and reproducible.

## CONCLUSION

The all-round AI-Chemist with a scientific mind reported here consists of a service platform, a mobile robot, workstations and a computational brain. It is able to read literature, to propose hypotheses, to design experimental plans, to execute automated operations, to analyse experimental data, to train machine-learning models and to feedback new hypotheses. In other words, it possesses all the abilities to conduct high-level chemical research, which can only be possible for a group of well-organized human chemists. The closed-loop iterative design of our AI-Chemist shows its versatility in the fields of electrocatalysts, photocatalysts and luminescent materials. The AI-Chemist possesses a universal software protocol and standardized hardware interface. This modular design makes it extendable to meet the requirements of various experimental tasks via adding more experimental workstations or computational mechanisms in the future. The fact that optimal materials could be intelligently screened out or designed by the AI-Chemist can certainly greatly reduce the time for human chemists to do experiments. It should also be mentioned that the AI-Chemist can only acquire information from the existing knowledge and conduct experiments within the known techniques. Future discovery still largely depends on human scientists to develop new theories and invent new technology. Nevertheless, it is safe to say that the AI-Chemist has begun to change our ways of finding and making new materials. All-round AI-Chemists with scientific minds may completely change the conventional chemical laboratory in the coming years.

## METHODS

### DFT calculation

The cluster of MOF is obtained based on the crystal structure of MIL-101. Subsequently, all the DFT calculations of the electrical structures are adopted by the Vienna ab-initio simulation package (VASP) [28]. Considering the long-range van der Waals (vdW) interaction corrections, the Grimme's DFT-D3 method is applied with an energy cut-off of 400 eV. The Perdew–Burke–Ernzerhof functional [29] is applied and the projector augmented wave method (PAW) [30] is implemented as the basis set for the plane-wave expansion. The convergence of forces and energies for the geometric structures are set to 0.01 eV/Å and 10^−5^ eV, respectively. The Brillouin zone is sampled with 1 × 1 × 1 Monkhorst–Pack k-mesh with a vacuum space of ∼15 Å.

### Calculation for the free energies

Based on the general single site of the OER mechanism, the four electron–proton transfer steps involved in the whole reaction could be written as:

(1) \begin{equation*} * +\, {{\rm{H}}}_2{\rm{O}}( {\rm{l}} ) \to {\rm{OH}}^* +\, {{\rm{H}}}^ + + {{\rm{e}}}^ - \end{equation*}

(2) \begin{equation*} {\rm{OH}}^* \to {\rm{O}}^* +\, {{\rm{H}}}^ + + {{\rm{e}}}^ - \end{equation*}

(3) \begin{equation*} {\rm{O}}^* +\, {{\rm{H}}}_2{\rm{O}}\left( {\rm{l}} \right) \to {\rm{OOH}}^* +\, {{\rm{H}}}^ + + {{\rm{e}}}^ - \end{equation*}

(4) \begin{equation*} {\rm{OOH}}^* \to * + {{\rm{O}}}_2 + {{\rm{H}}}^ + + {{\rm{e}}}^ - \end{equation*}

To calculate the free energy changes, a standard change of Gibbs free energy at zero potential was calculated according to the following equation:

$$\begin{eqnarray*}
\Delta {\rm{G}} = \Delta {\rm{E}} + \Delta {\rm{ZPE}} - {\rm{T}}\Delta {\rm{S}},
\end{eqnarray*}$$

where ΔE refers to the change in reaction energy based on DFT simulations, ΔZPE is the zero-point energy change calculated by the vibrational frequency using the finite difference method and ΔS represents the entropy change for each elementary step. The temperature in our work is set to be 298.15 K.

### Molecular dynamics simulations

Classical molecular dynamics (CMD) simulations are performed using the LAMMPS package [31] in the isothermal-isobaric (NPT) ensemble via the Nose-Hoover barostat and thermostat [32,33]. To accelerate the structural sampling, the CMD simulations are run at 3000 K with a time step of 1 fs. The initial simulation box of 4 × 4 × 4 nm^3^ has periodic boundaries in the all directions. Sixty different metal atoms, 20 oxygen atoms and 40 terephthalic acid ions are randomly placed into the box. The force-field parameters of them are all generated using the LAMMPS Interface program [34]. The cut-off distance for the Lennard–Jones and coulombic potential is 12.5 Å. The duration of the simulation is 1 ns.

### Expertise rules for AIE luminophores data cleaning

To identify the practically available AIE luminophores, several expertise rules are applied. First, the CTE molecules and common solvents are removed, leaving 306 molecules. Then, two more criteria are applied to further shorten the candidate list: water solubility and frequency of fluorescence light. The top entry is tetraphenylethylene, a well-known and probably the most studied organic AIE dye [35–37]. An ML model is trained to predict the water solubility of all the molecules based on their SMILES. After removing those that are expected to be insoluble or sparsely soluble in aqueous solution, the list reduces to 20 s. The fluorescence frequencies of each of them are predicted by calculating the energy gap between LUMO and HOME using DFT. Only molecules with fluorescence located in the visible range make the final list. The top entry is berberine (or berberine chloride).

### Neural network architecture

The first neutral network (NN) applied different metal ratios as descriptors to predict the catalytic properties (GOH^*^, GO^*^–OH^*^, Δe). To obtain the relations between catalytic properties and real overpotential, the second NN with the catalytic properties as descriptors was implemented to predict the real overpotential. One input layer, three hidden layers and one output layer constituted the whole NN. The number of neurons in the hidden layer was 128, 128 and 128, respectively. To establish the correlation between the descriptors and prediction targets, the data set (80% for training and 20% for validation) was trained using the Rectified Linear Unit activation function [38]. The dropout parameter [39] was used to prevent overfitting of the second NN. The backpropagation algorithm implemented in TensorFlow [40] enabled it to track the weights successfully and update them rapidly.

## Supplementary Material

nwac190_Supplemental_File
