# Supplementary material for: An all-round AI-Chemist with a scientific mind
Source: Natl Sci Rev. 2022 Sep 8;9(10):nwac190. doi: 10.1093/nsr/nwac190 (PMC9674120; doi:10.1093/nsr/nwac190)
Supplement: nwac190_Supplemental_File [file nwac190_supplemental_file.zip › SI_Proof.docx]

*Supporting Information for*

**An All-Round AI-Chemist with Scientific Mind**

Qing Zhu^1,†^, Fei Zhang^2,†^, Yan Huang^1,†^, Hengyu Xiao^1,†^, LuYuan Zhao^1^, XuChun Zhang^2^, Tao Song^2^, XinSheng Tang^2^, Xiang Li^2^, Guo He^2^, BaoChen Chong^2^, JunYi Zhou^2^, YiHan Zhang^2^, Baicheng Zhang^1^, JiaQi Cao^1^, Man Luo^1^, Song Wang^1^, GuiLin Ye^3^, WanJun Zhang^3^, Xin Chen^3^, Shuang Cong^2^, Donglai Zhou^1^, Huirong Li^1^, Jialei Li^1^, Gang Zou^1^, WeiWei Shang^2,*^, Jun Jiang^1,4,*^, Yi Luo^1,4,*^

^1^ Hefei National Research Center for Physical Sciences at the Microscale, School of Chemistry and Materials Science, University of Science and Technology of China, Hefei 230026, China

^2^ School of Information Science and Technology, University of Science and Technology of China, Hefei 230026, China

^3^ Hefei JiShu Quantum Technology Co. Ltd., Hefei 230026, China

^4^ Hefei National Laboratory, University of Science and Technology of China, Hefei 230088, China.

^*^ Corresponding authors. E-mails: wwshang@ustc.edu.cn; jiangj1@ustc.edu.cn; yiluo@ustc.edu.cn)

^†^ Equally contributed to this work.

# Home-Developed System Software

**Figure S1.** System software framework of the AI-Chemist. The mobile robot consists of a Ridgeback omnidirectional mobile platform from Clearpath Robotics and an UR5e robotic arm from Universal Robots. It is equipped with an Intel RealSense D435i depth camera and a PGI-140 two-finger gripper from DaHuan Robotics. The self-developed system software framework of the mobile robot is shown in the green box, which mainly includes a perception and manipulation module, a navigation and planning module, and a manipulation task management module. The control system software of the mobile robot is developed based on the Robot Operating System (ROS) platform. Each functional module in ROS uses UDP protocol to communicate with each other and uses TCP protocol to communicate with the smart chemistry workstations and backstage management system.

# Service Platform with Web-Based Interface

**
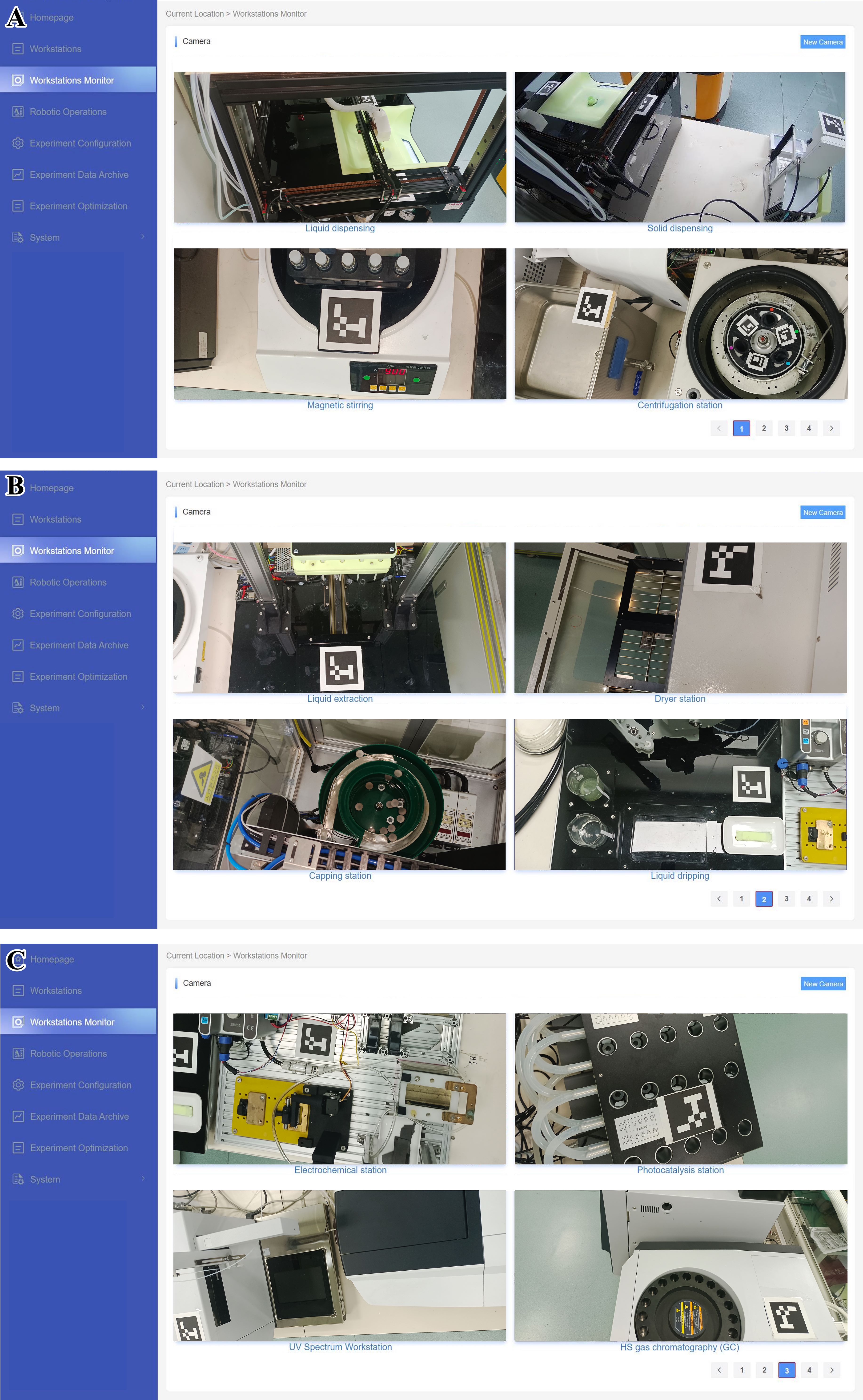
**

**Figure S2.** The interface for monitoring workstations. (A) liquid dispensing, solid dispensing, magnetic stirring, centrifugation station. (B) liquid extraction, dryer station, capping station, liquid dripping. (C) electrochemical station, photocatalysis station, UV spectrum/fluorescence spectroscopy workstation, HS gas chromatography (GC).

**
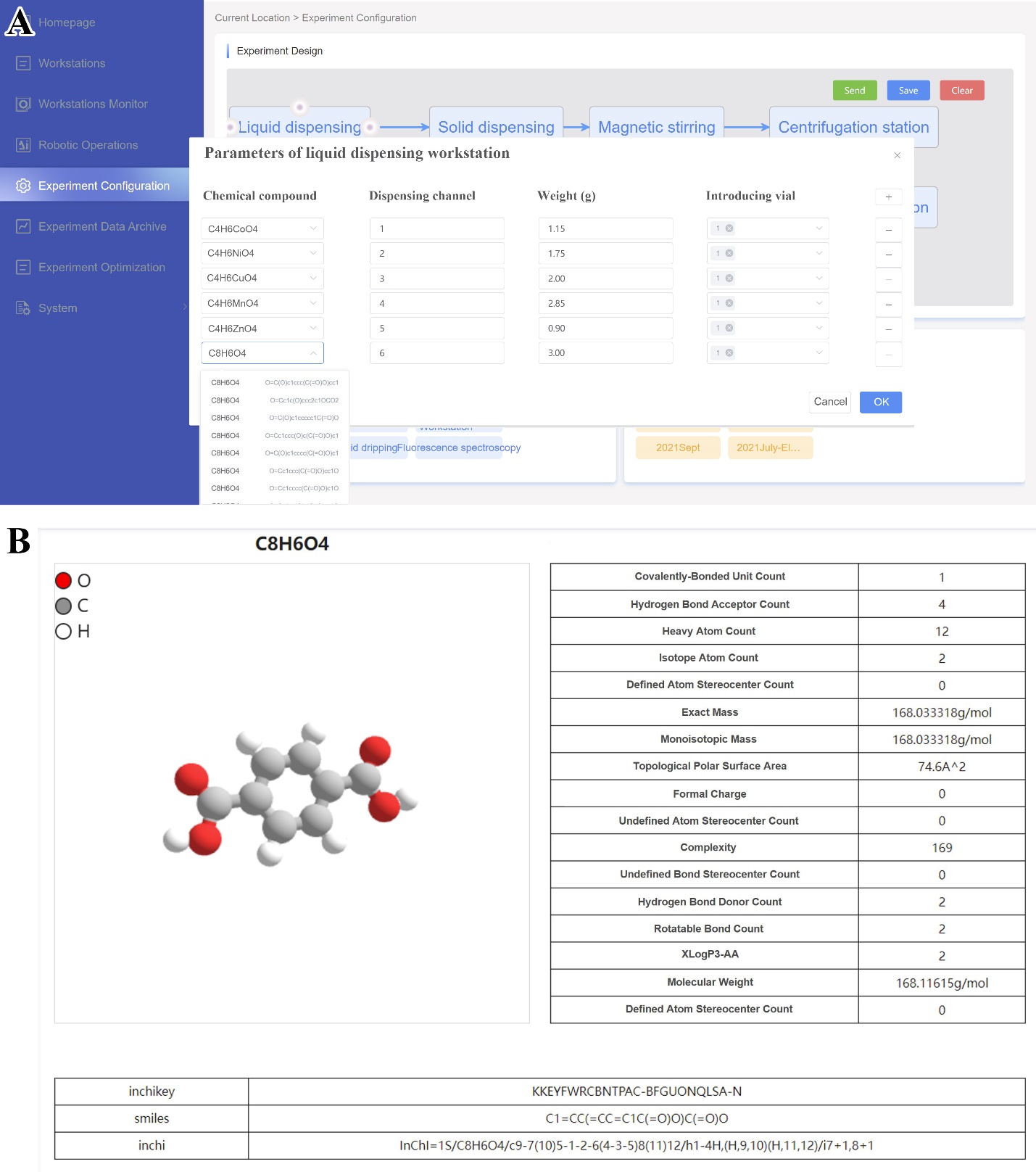
**

**Figure S3.** The service platform GUI and database of chemical compounds. (A) GUI for choosing chemical samples from the solid or liquid dispensing menu. (B) The properties of the compounds (such as terephthalic acid) can be queried through a web interface.

**
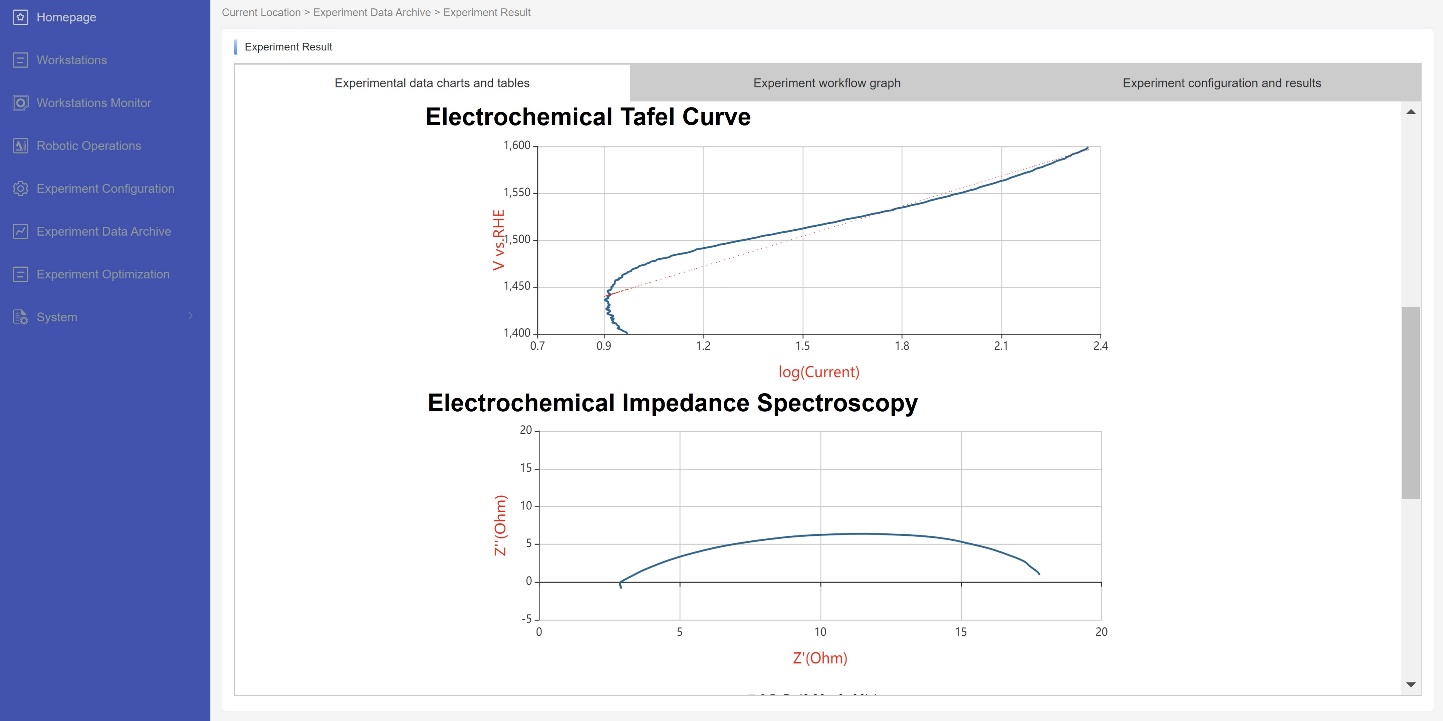
**

**Figure S4.** Data display interface for electrochemical Tafel curve and electrochemical impedance spectroscopy.

**
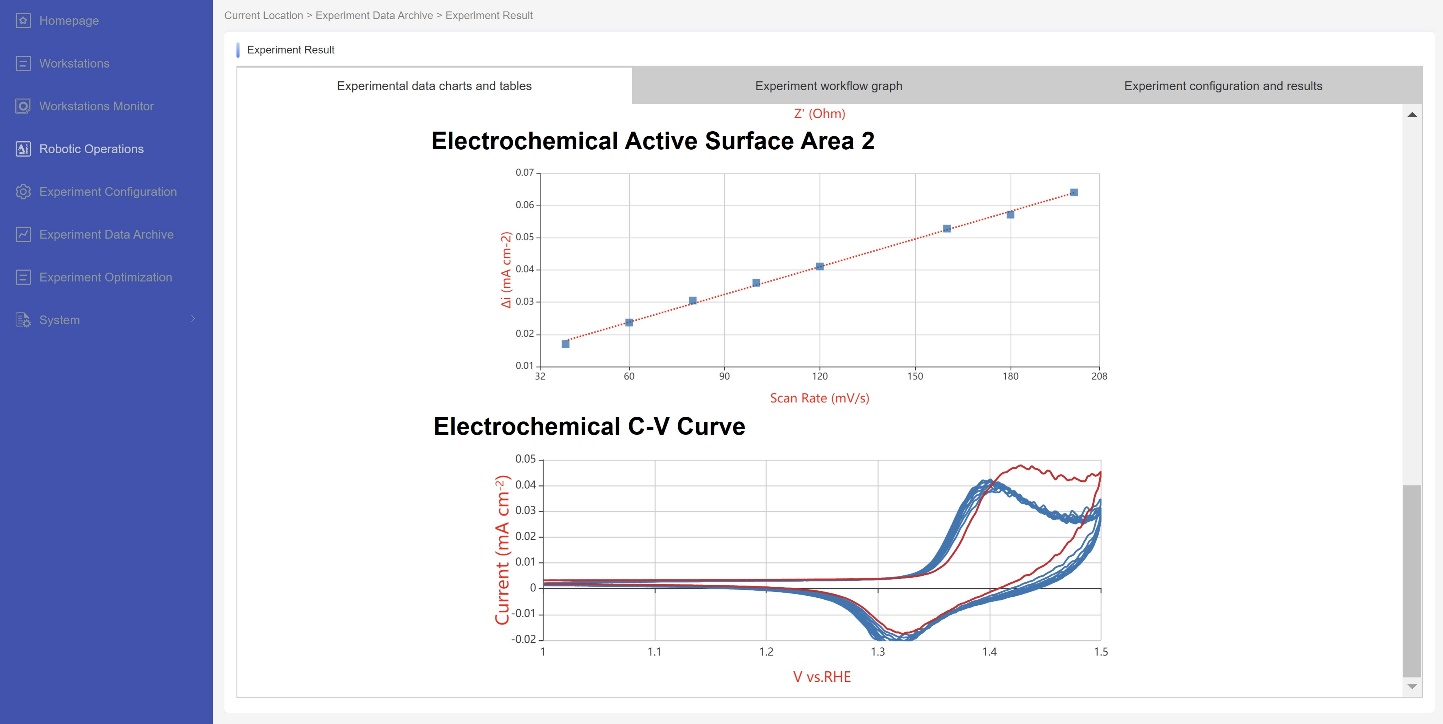
**

**Figure S5.** Data display interface for electrochemical active surface area and electrochemical current-voltage (C-V) active curve.

**
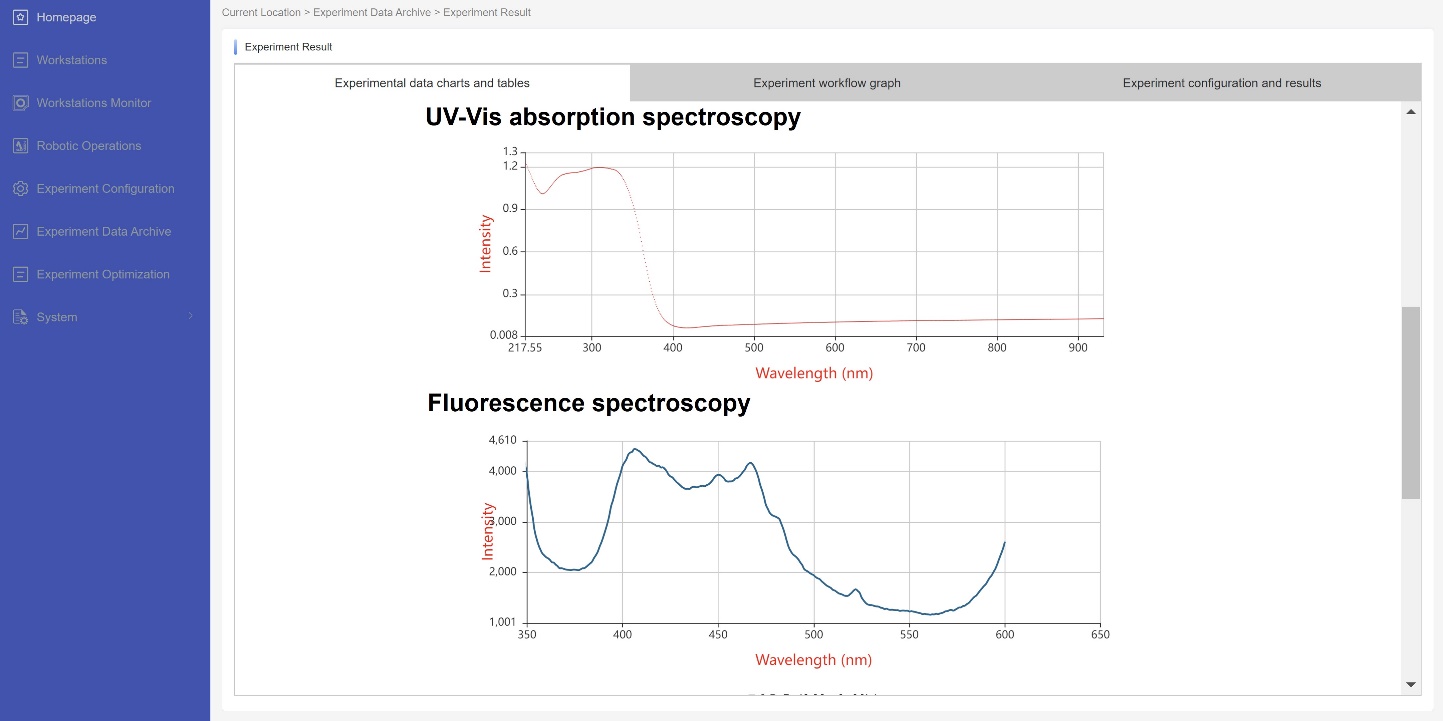
**

**Figure S6.** Data display interface for UV-Vis absorption and fluorescence spectroscopy.

**
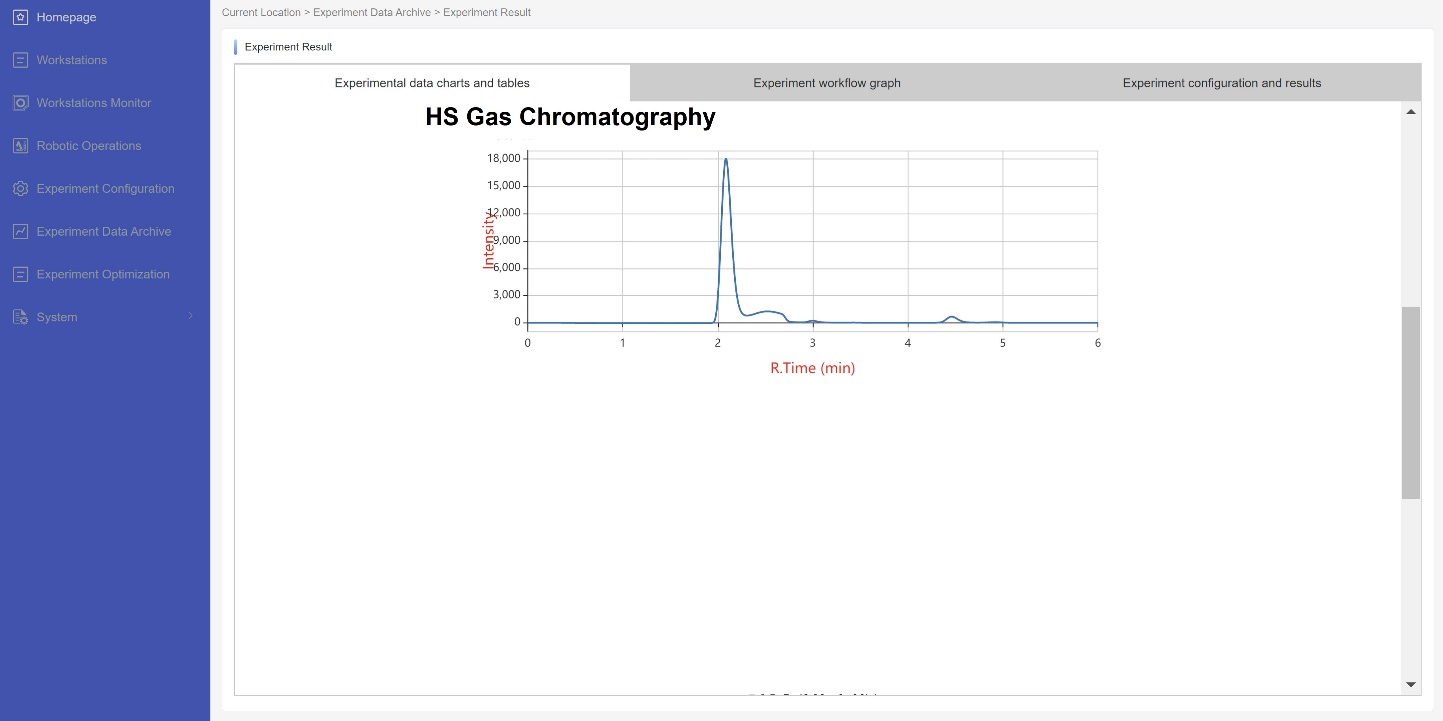
**

**Figure S7.** Data display interface for HS gas chromatography.

**
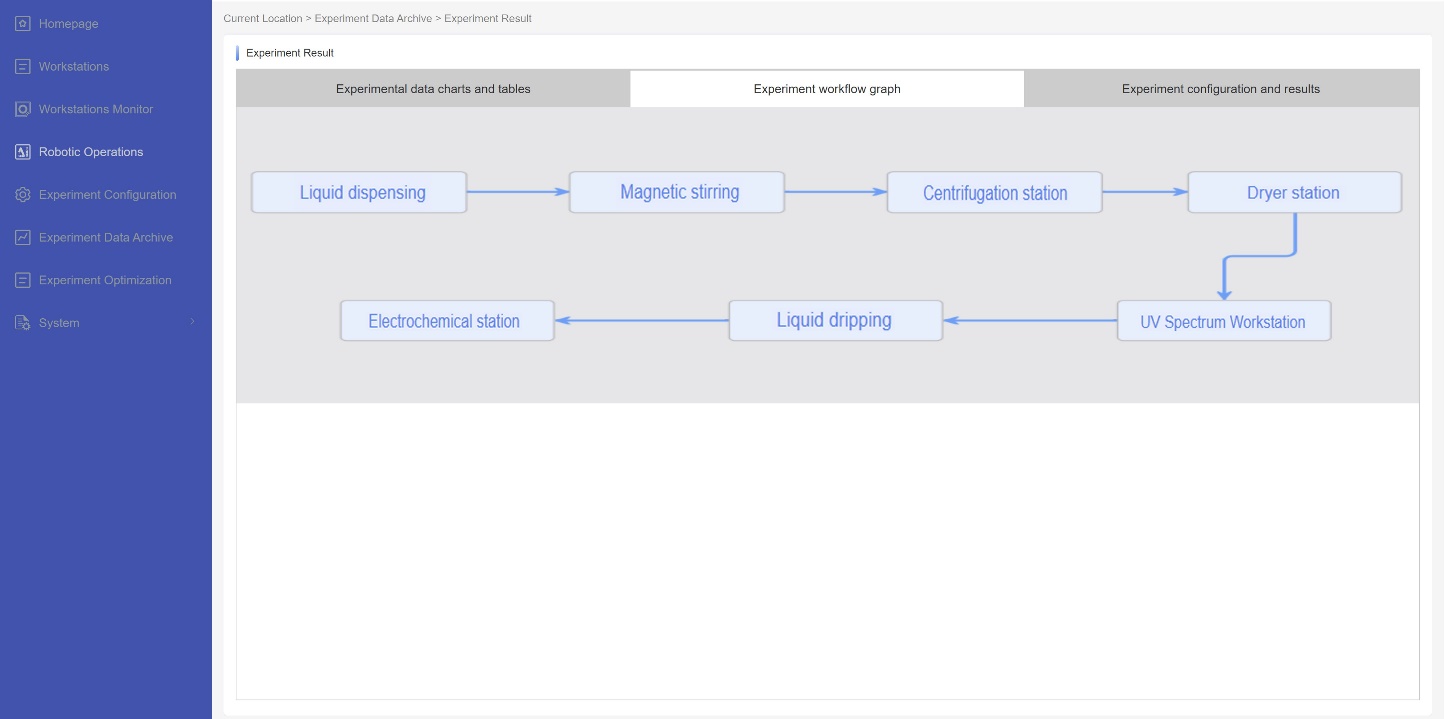
**

**Figure S8.** Experimental workflow diagram of a typical electrocatalytic experiment.

**
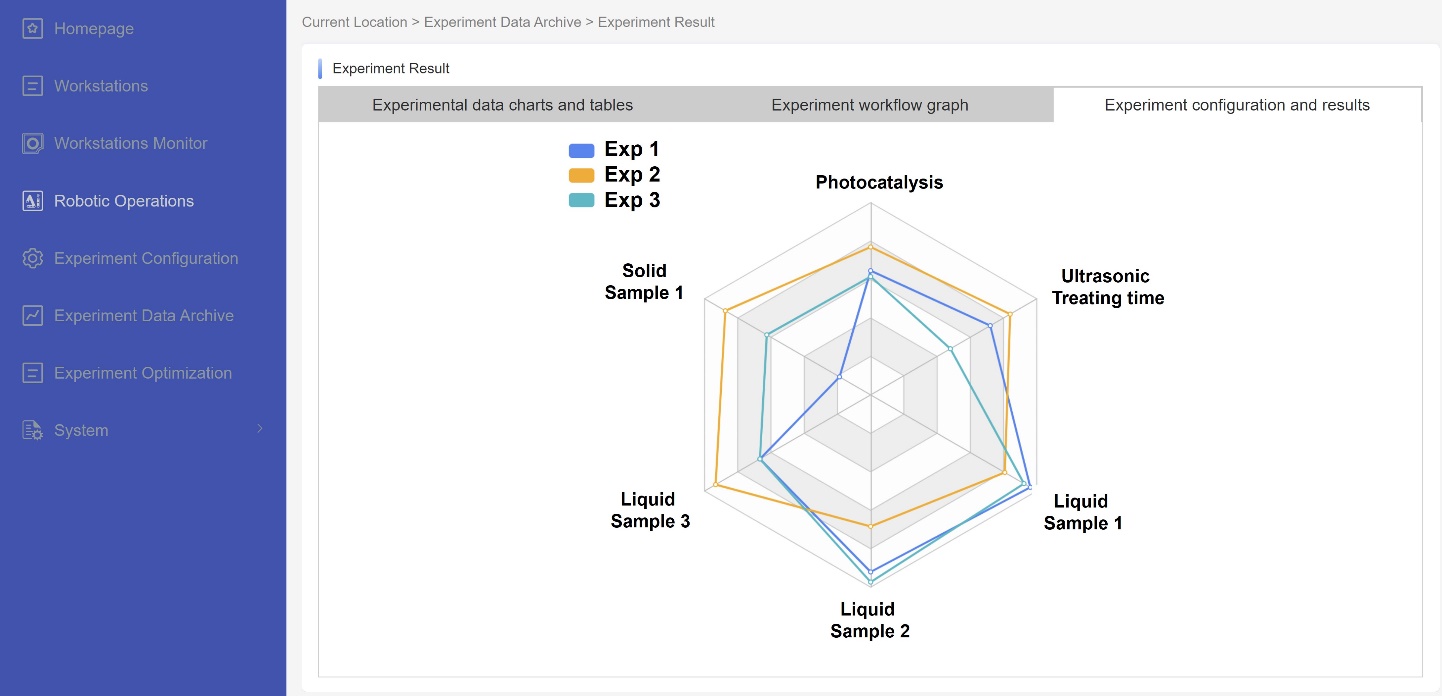
**

**Figure S9.** Experimental conditions graph of a typical photocatalytic experiment.

**
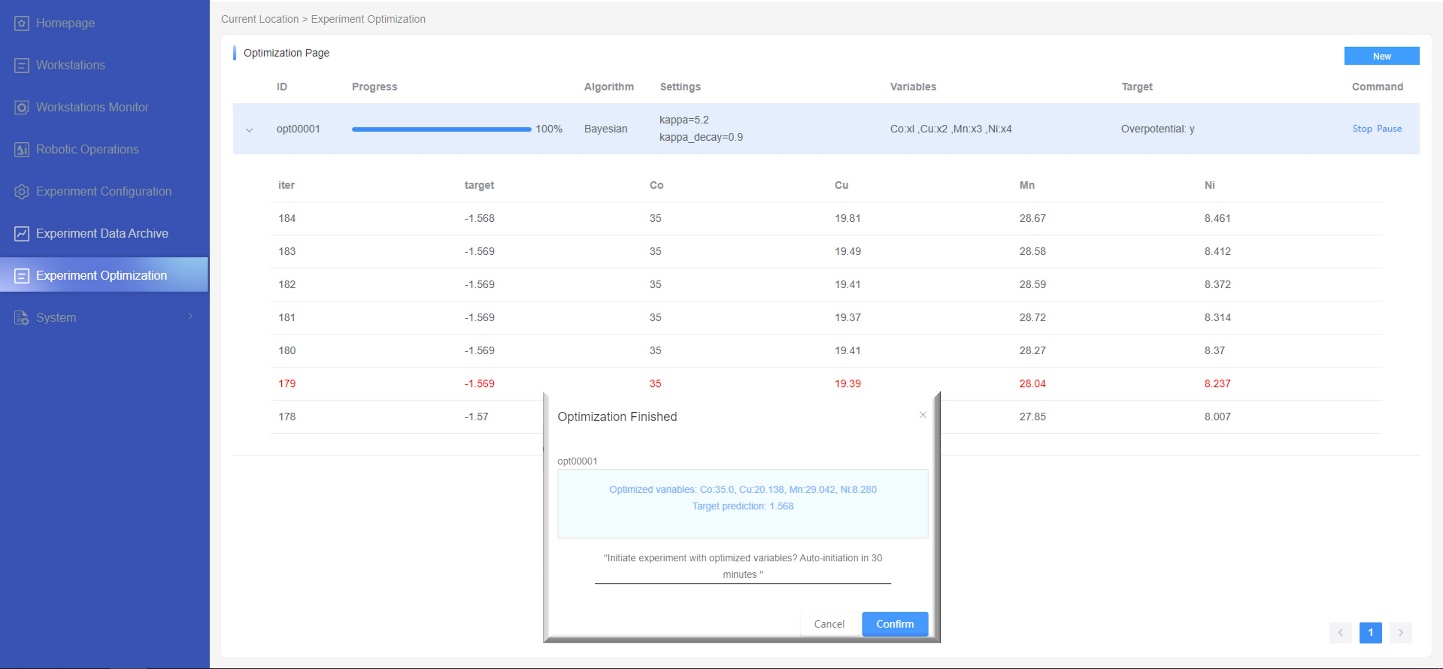
**

**Figure S10.** Bayesian optimization processing during a typical electrocatalytic experiment.

# Smart Chemical Workstations

**
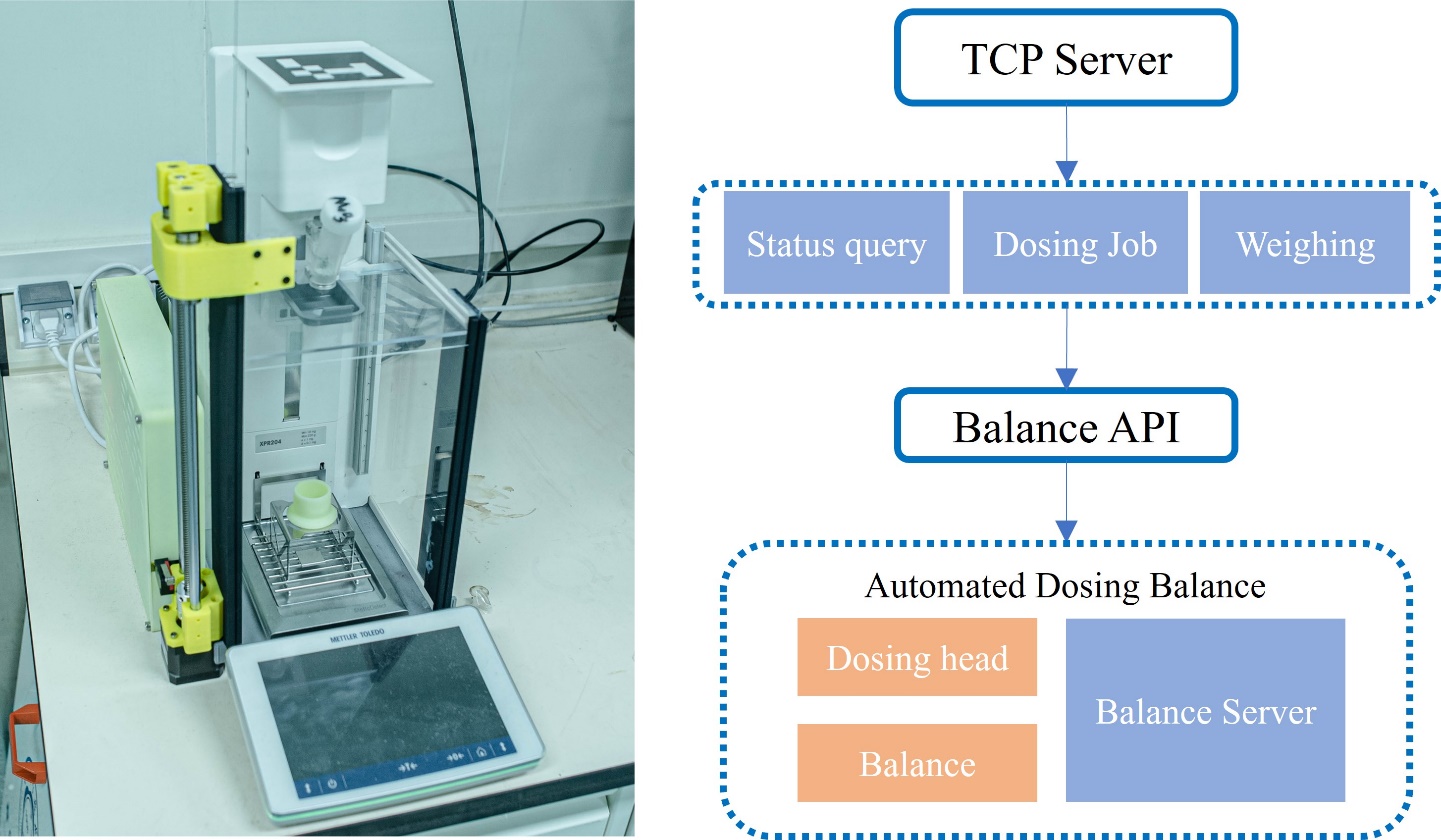
**

**Figure S11.** Solid dispensing workstation. The solid dispensing station is converted from a commercial equipment. Its main body is an automatic sampling balance (METTLER TOLEDO XPR204/A), which can realize the operation of adding powdered solid materials with an accuracy of 0.1 mg to a standard sample bottle. Its windshield was retrofitted into an automatically switchable accessory to allow robotic arm access the vials without sacrificing accuracy. The manufacturer provides a web service interface based on the Simple Object Access Protocol (SOAP). A self-developed communication software controls the solid sampling balance in real time. The operations that can be completed include state detection, weighing, sample addition, and setting allowable errors.

**
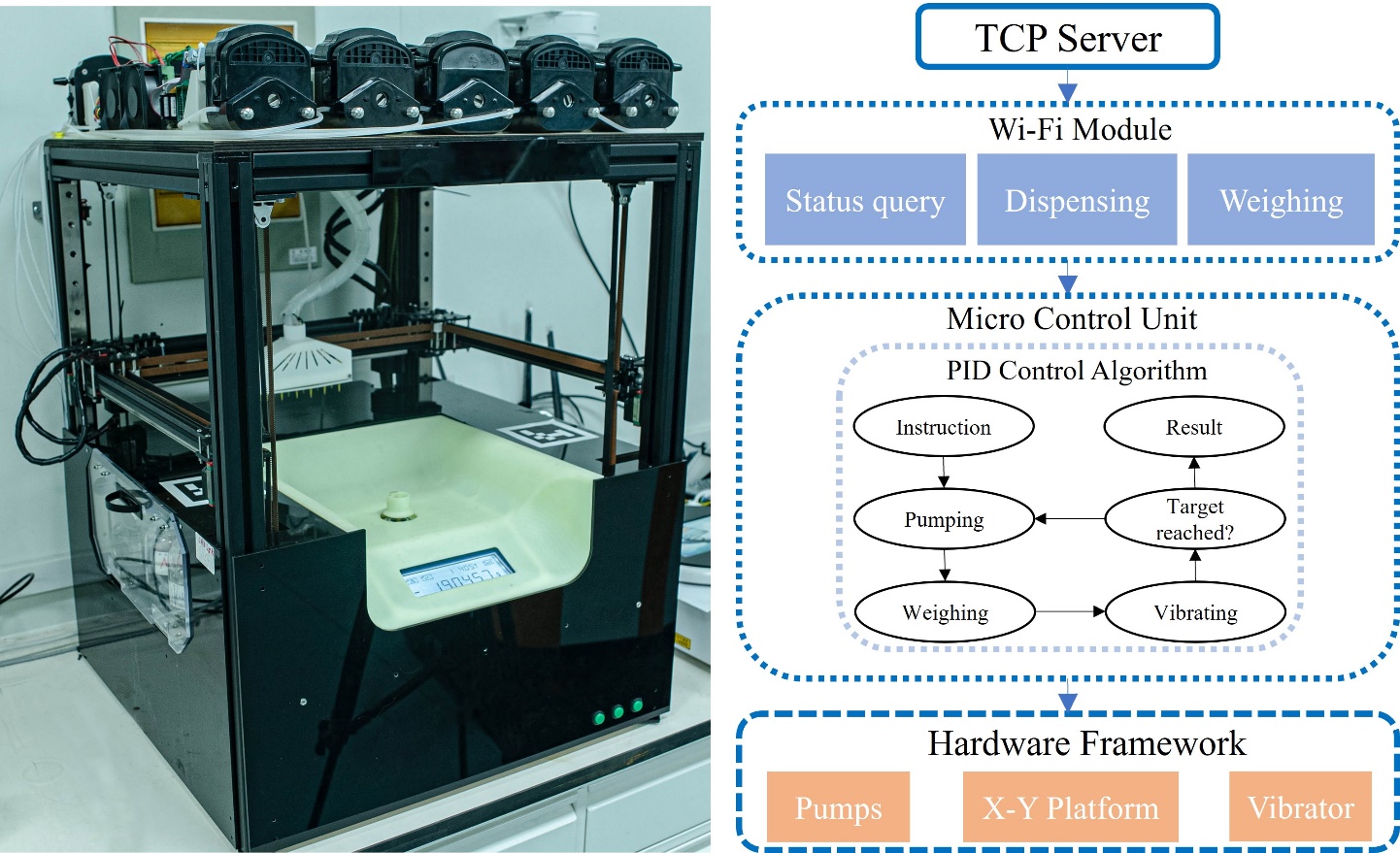
**

**Figure S12.** Liquid dispensing workstation. The liquid dispensing workstation is a self-developed equipment. The control chip (MEGA2560) controls the injection function of 12 peristaltic pumps (Chuangrui BJ100M/YZ1515X) for different liquids, and a wireless communication module (ESP8266) is installed to support the network communication protocol. The output line of each pump is installed in a different position on the dosing head (manufactured by 3D printing). The workstation uses a self-built mobile platform (using a 57HBP76AL4-TFA stepper motor as the power source) to adjust the sampling position, and cooperate with the pump body to switch the liquid sampling channel. An electronic balance with readability of 0.1 mg (Lichen Technology FA124) is installed below to feedback the sample addition quality. The Proportional-Integral-Derivative (PID) algorithm receives the real-time quality data, so as to control the peristaltic pump to adjust the sampling speed. Therefore, the sampling accuracy can reach 3 μL.

**
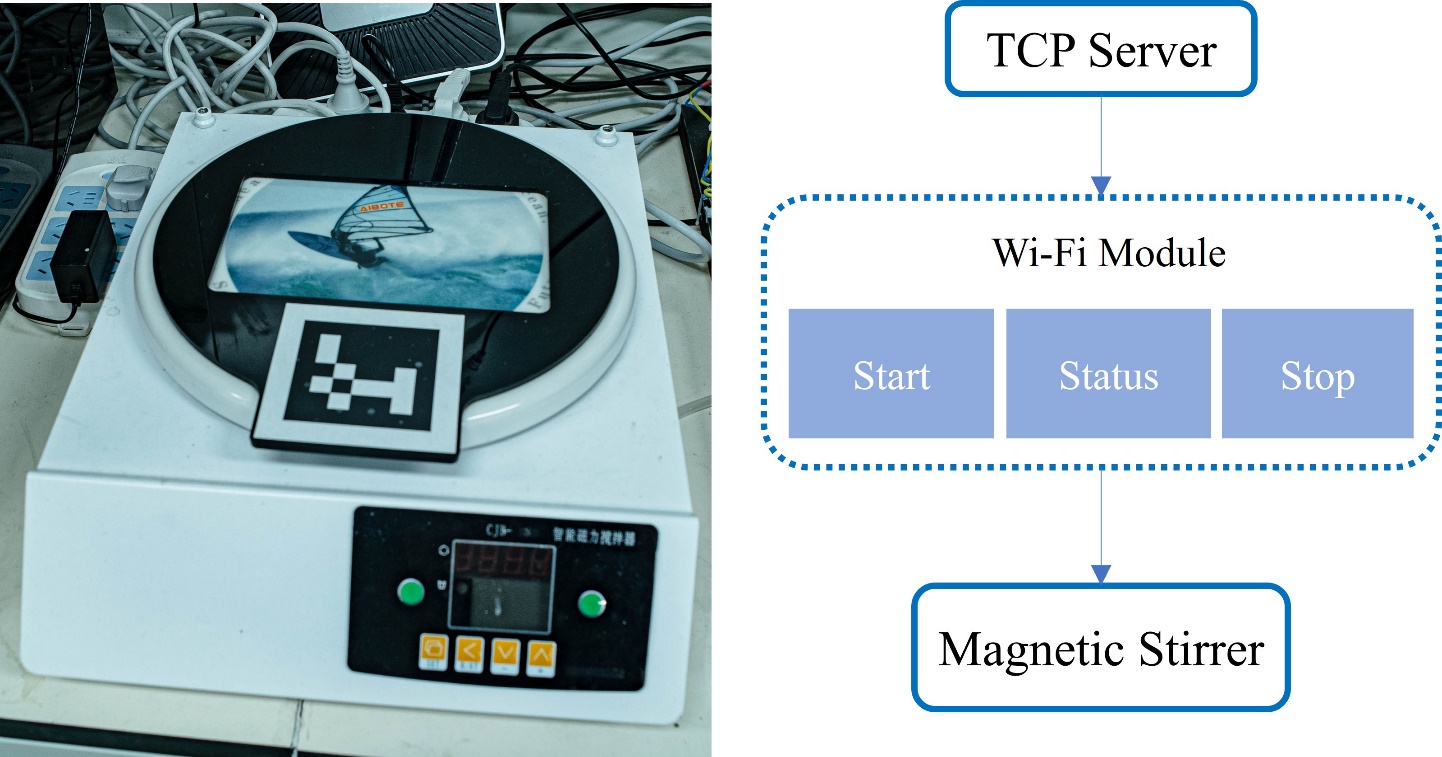
**

**Figure S13.** Magnetic stirring workstation. The magnetic stirring workstation is converted from a commercial device. Its main body is a magnetic stirrer (CJB-S intelligent magnetic stirrer). A limiting slot plate is placed on the stirring platform to limit the placement position of vials by the robot. The workstation is capable of stirring multiple standard vials and heating up to 350 °C with accuracy of ±1 °C at the same time. The workstation is modified to automatically control its startup and shutdown, so that the workstation has added the functions of timing on, timing off, and stirring time feedback, which realizes the precise control of the stirring time in milliseconds.

**
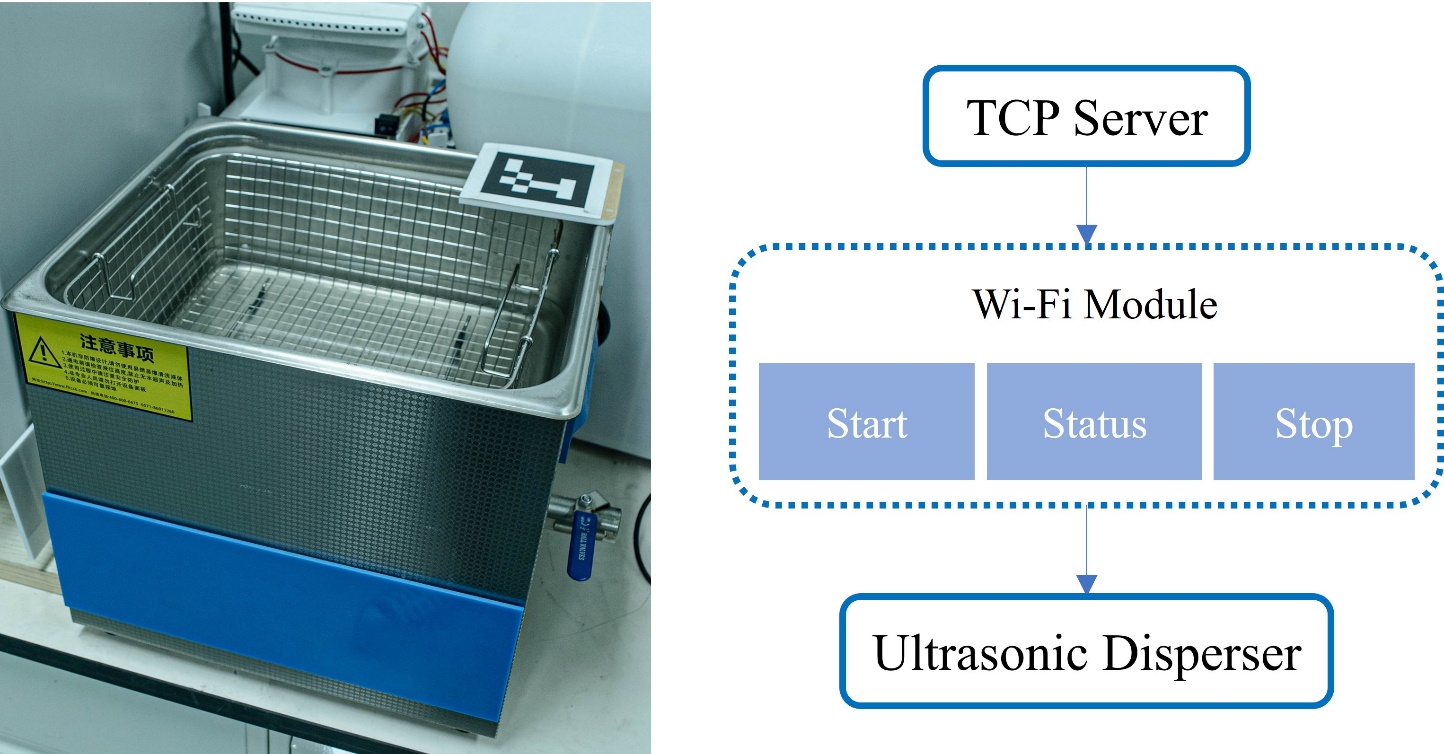
**

**Figure S14.** Sonication mixing workstation. The sonication mixing workstation is converted from a commercial device (FRQ-1006 ultrasonic cleaner) and added to the network control. The device can ultrasonically disperse samples in 10 sample vials at the same time. The light-current control line of its host is connected to a wireless communication Micro Controller Unit (MCU, ESP8266) to change its working state, realizing the precise control of the ultrasonic dispersion time in milliseconds. A drying device (MODUN M2008C) is installed behind the ultrasonic tank to dry the sample vials. The switch control of the drying equipment is also connected to the MCU to coordinate the working sequences of the ultrasonic dispersion process.

**
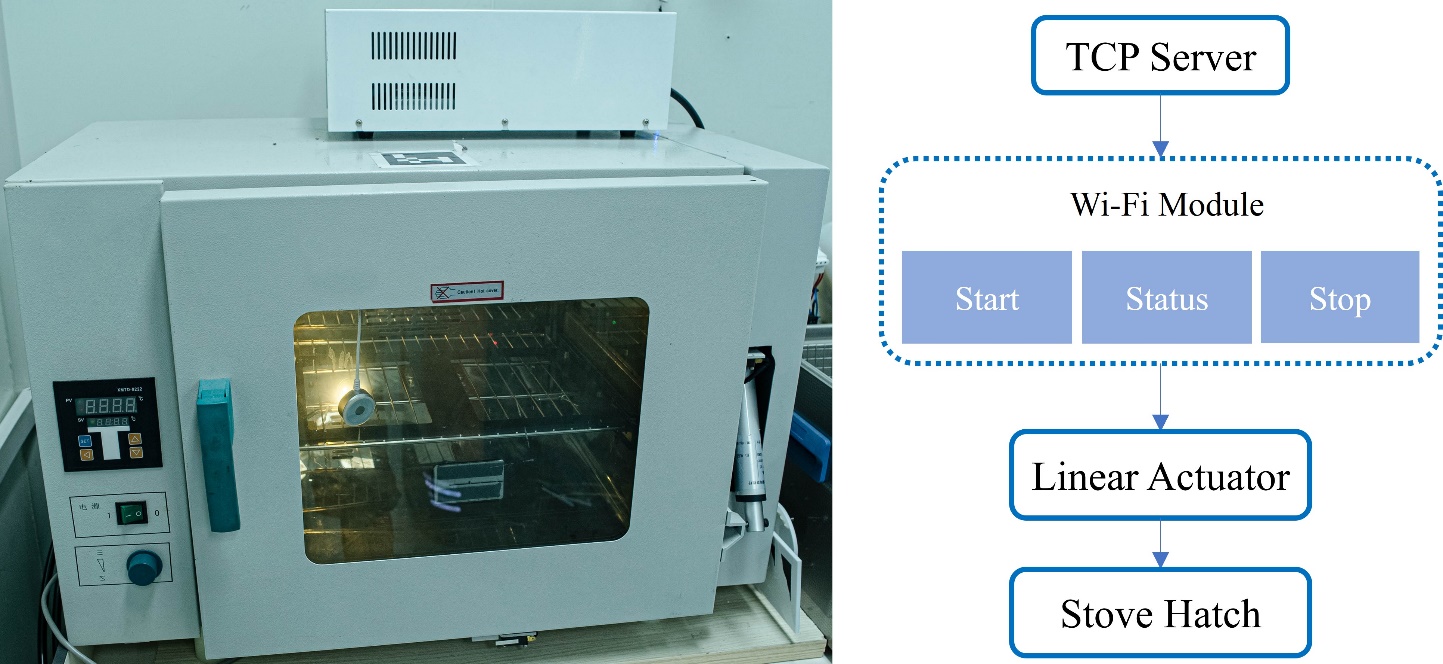
**

**Figure S15.** Dryer workstation. The dryer workstation is a commercial equipment (Jinghong XMTD-8222) which has been modified in power and structure. It can simultaneously dry 20 standard sample vials within adjustable temperature range. Through the modification of the mechanical structure, when the door is opened, the drying bracket can automatically extend outwards, providing space for the robotic arm to operate the sample vials. Two sets of positioning grooves are fixed on the drying rack to avoid the positional deviation of the robotic arm caused by the movement of the rack affecting the position of the sample vial. The hatch is equipped with a power shaft to realize automatic opening and closing. Under the control of the wireless module (ESP8266), the power shaft can remotely accept the command to open and close the hatch.

**
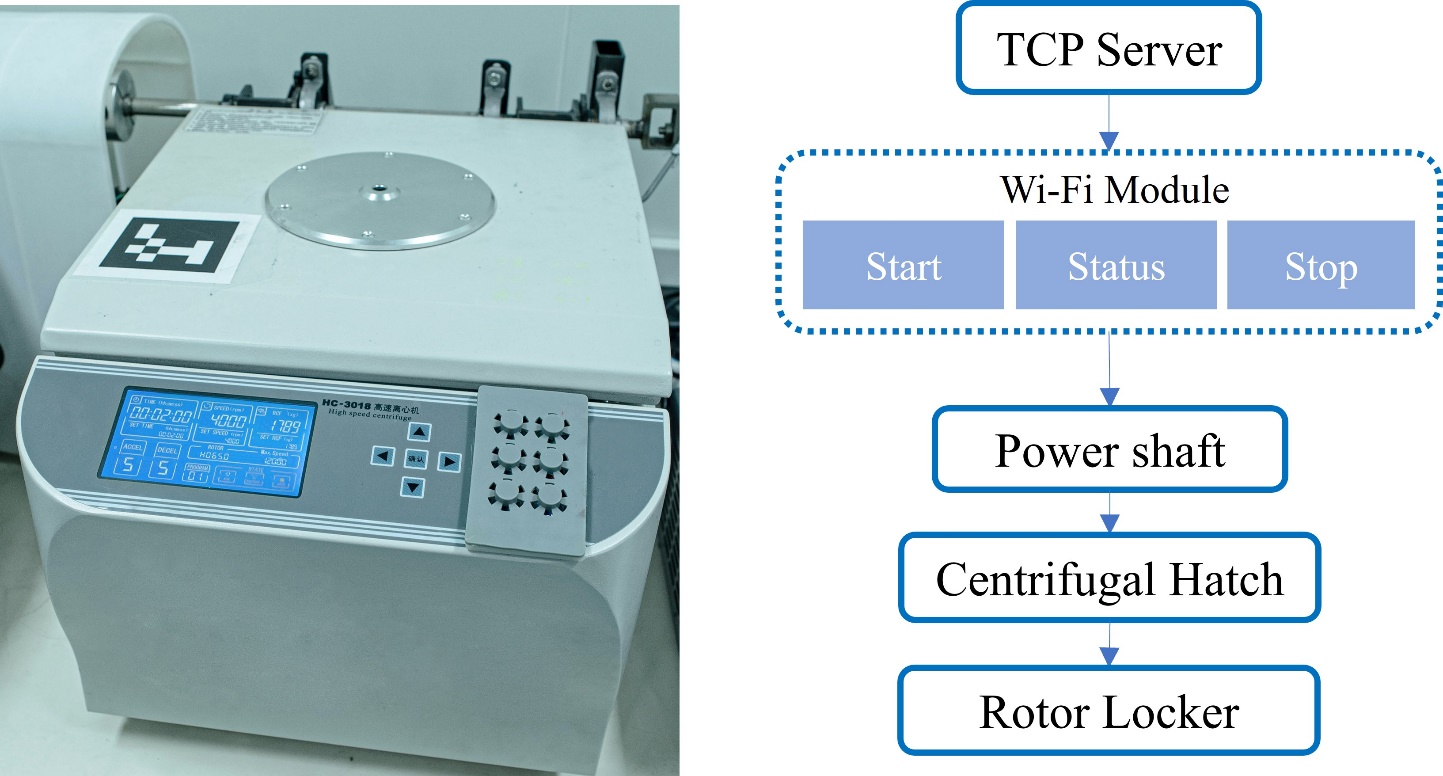
**

**Figure S16.** Centrifugation workstation. The centrifugation workstation is a commercial equipment (Zhongjia HC-3018 high-speed centrifuge) that has been transformed with power. It can automatically perform high-speed centrifugation on three standard sample vials at the same time, and the maximum speed can reach 12000 r/min. The vial can be firmly inserted into the centrifuge hole through the sleeve (manufactured by 3D printing). The installed power shaft realizes the automatic opening and closing of the centrifuge cover. Under the control of the wireless module (ESP8266), the power shaft can remotely accept the command. The rotor of the centrifuge is equipped with a brake drum. When the centrifuge cover is opened, the brake drum can lock the rotor part of the centrifuge, so that the robotic arm can be aligned with the hole of the centrifuge.

**
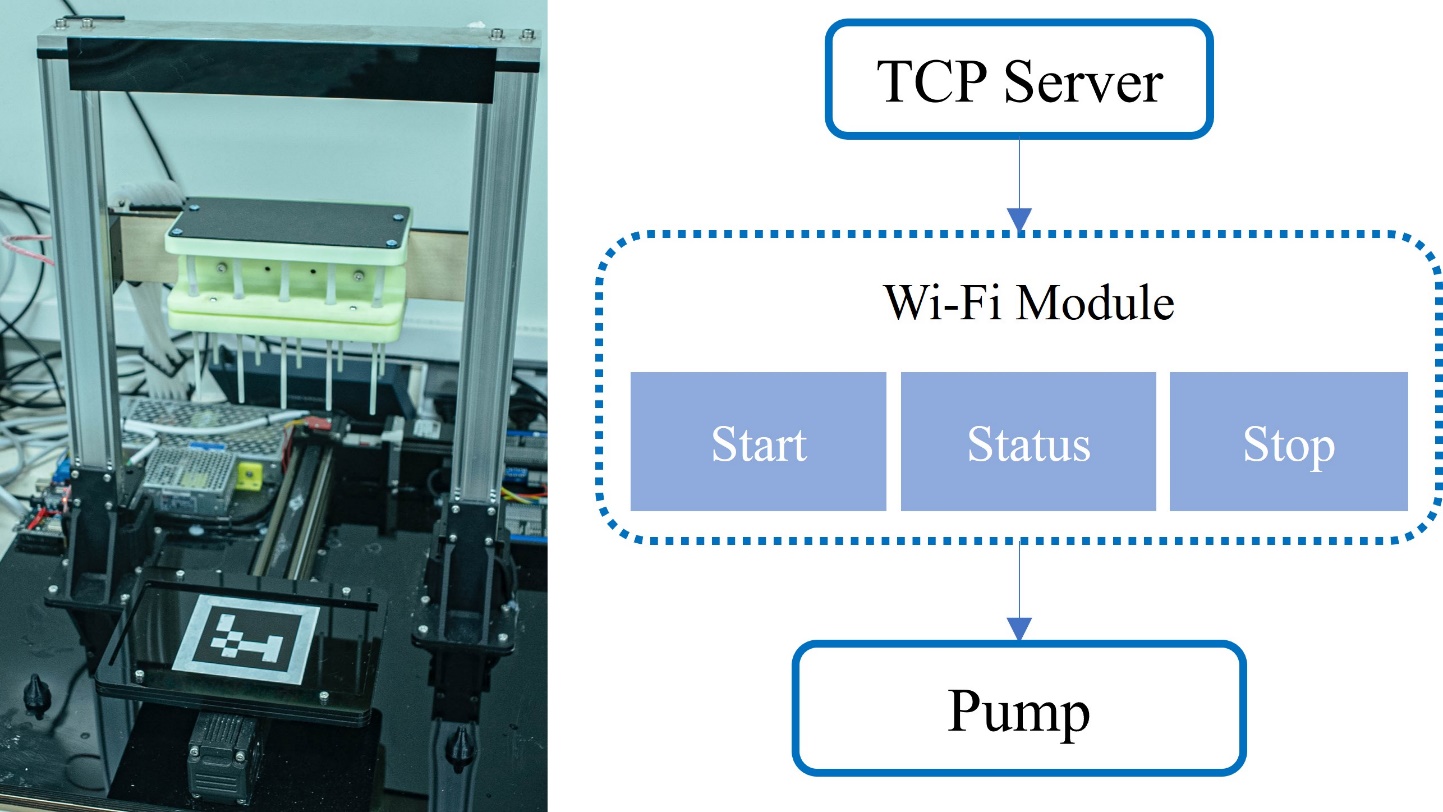
**

**Figure S17.** Liquid extraction workstation. The liquid extraction workstation is a self-developed device. Through the wireless module (ESP8266), it directly controls 10 high-pressure water pumps (DHE-755C), which can simultaneously extract the supernatant from 10 standard vials after centrifugation. The sample vial enters and leaves the extraction position through a self-built moving platform (using a 57HBP76AL4-TFA stepper motor as the power source) to provide space for the robotic arm to operate on standard sample vials.

**
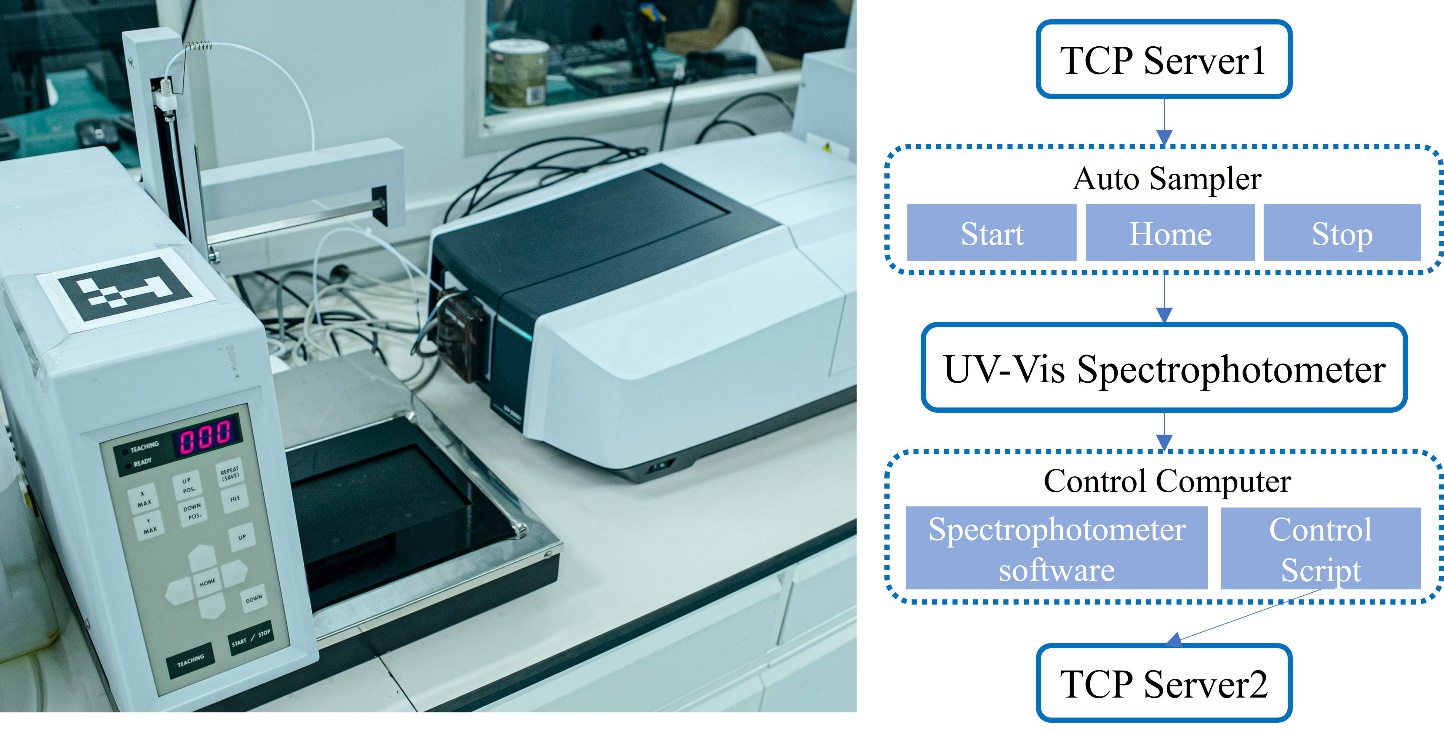
**

**Figure S18.** UV-Vis spectroscopy workstation. The UV/Vis spectroscopy workstation includes a commercial UV spectral analyzer (Shimadzu UV-2600i), a modified autosampler (Shimadzu ASC-5), and a computer. The workstation can automatically and continuously measure UV/Vis spectrums. The workstation is equipped with the wireless module (ESP8266), which can accept commands through the network and fully automated inject samples. A self-developed software, which controls the spectroscopy software to complete the characterization operation by simulating mouse and keyboard, can autonomously acquire spectral data for preliminary analysis, form a graph, and transmit it to the service platform.

**
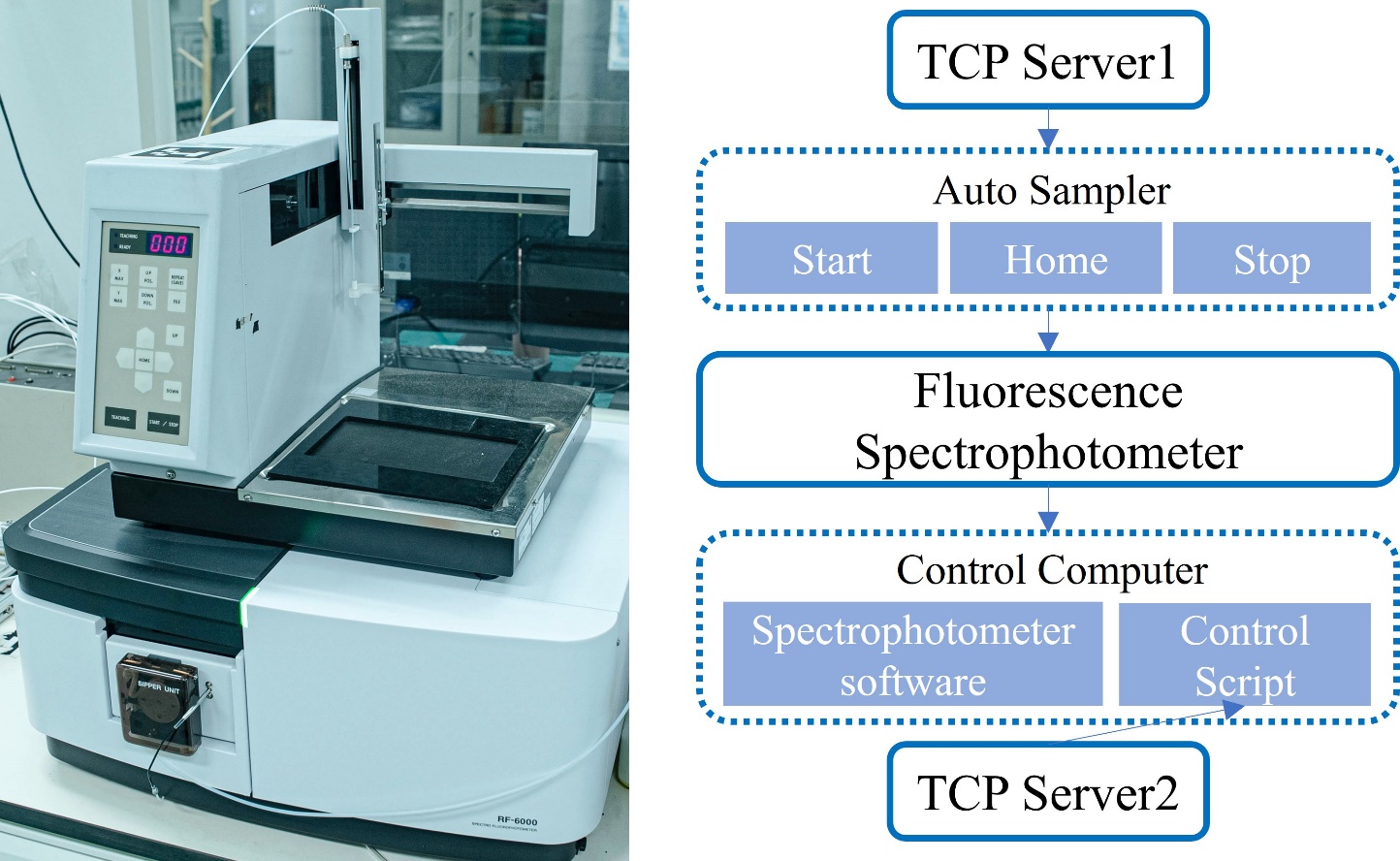
**

**Figure S19.** Fluorescence spectroscopy workstation. The fluorescence spectroscopy workstation includes a commercial fluorescence spectroscopy analyzer (Shimadzu RF-6000), a modified autosampler (Shimadzu ASC-5), and a computer. The workstation can automatically and continuously measure the fluorescence spectrums. The modification of the autosampler in this workstation and the self-developed software are the same as that for UV/Vis absorption spectroscopy workstation.


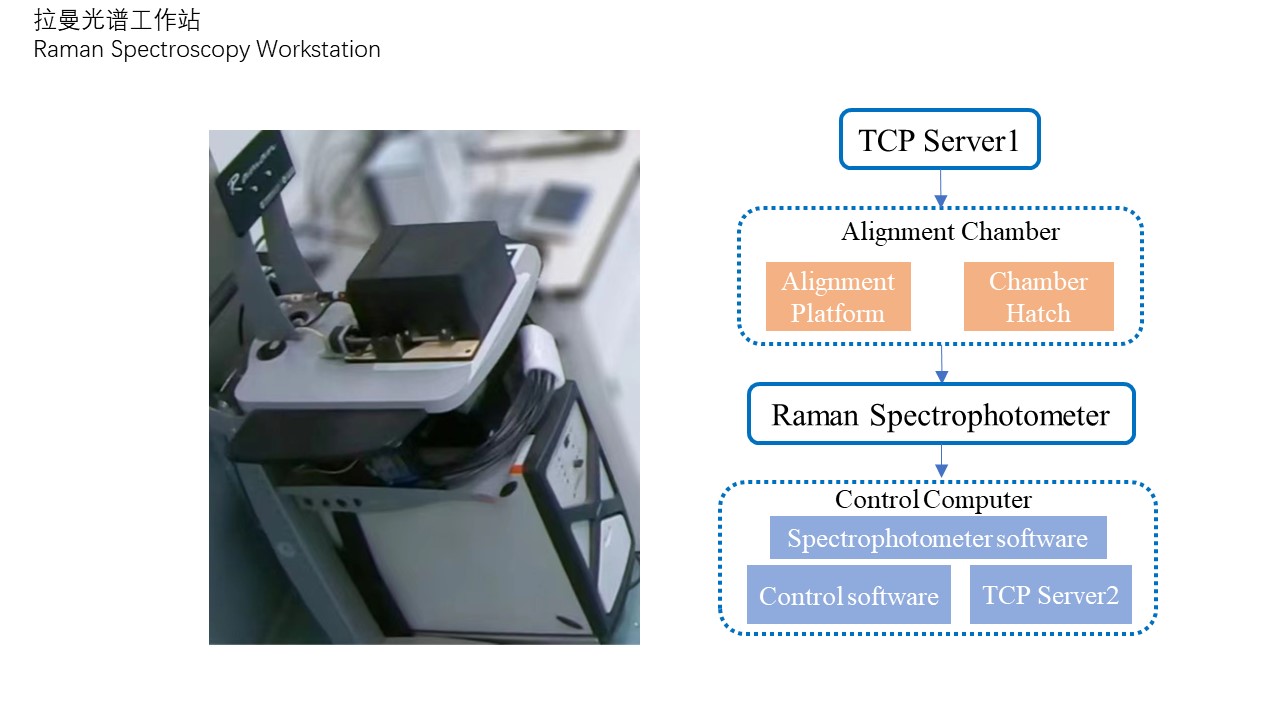


**Figure S20.** Raman Spectroscopy workstation. The Raman Spectroscopy workstation consists of a commercial Raman Spectroscopy analyzer (Kaiser, RXN2), a home-made alignment chamber and a controlling computer. The sample alignment platform (NuAES, A0-145c1-57-100ps3-201-A) allows adjusting the sample position to maximize Raman signals. Both the alignment platform and chamber hatch are controlled by wireless module (ESP8266) to receive instructions remotely. The supporting instrument software is home-developed. and is similar to that for UV/Vis absorption spectroscopy workstation.

**
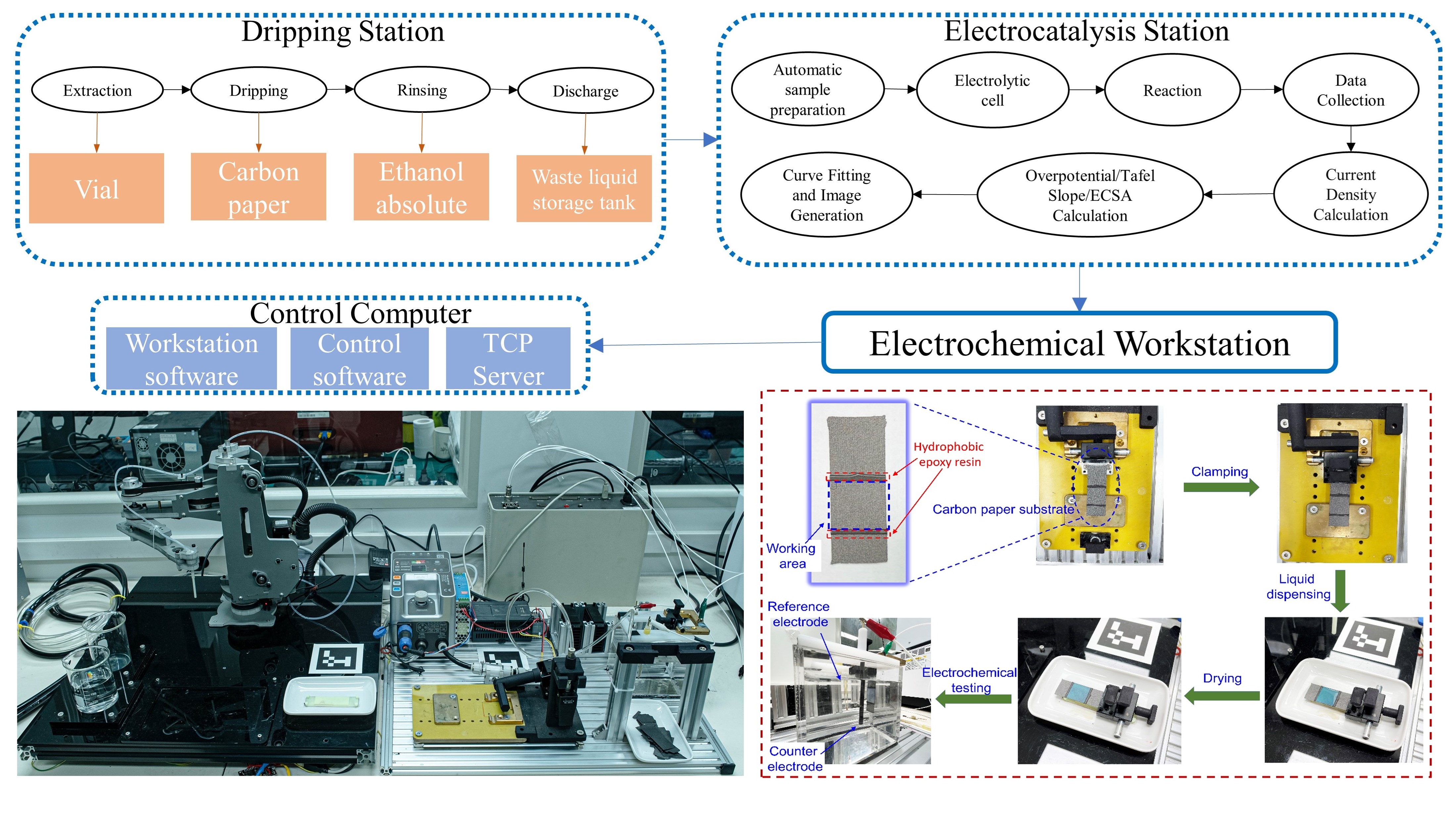
**

**Figure S21.** Electrochemical workstation. The electrocatalytic workstation consists of three equipments, including an electrocatalytic reaction platform, an electrocatalytic equipment, and a computer, which can test the electrocatalytic performance of the sample. The electrocatalytic reaction platform is operated by the robot, which can grasp the substrate carrying the sample through the pneumatic gripper, and then transport it to the electrochemical reaction cell by the robotic arm for testing. After the test, the robotic arm takes it out to the recovery position, releases the substrate, and resets the pneumatic gripper to complete the operation cycle. The electrocatalytic equipment is an electrochemical workstation (CHI-660E, CH Instrument). Its electrodes are connected to the reaction cell of the electrocatalytic reaction platform. The computer controls the electrocatalytic equipment through a self-developed software, and provides network services to accept remote commands. At the same time, the software can perform preliminary analysis and processing on the data obtained by the electrocatalytic equipment, and send the data back to the service platform. There is a self-developed auxiliary device for drip sample preparation, the main body of which is a small robotic arm (stepping four-axis Selective Compliance Assembly Robot Arm (SCARA)), which is equipped with an automatic extraction/drip device. Within the operating range of the robotic arm, there are four operating positions: sample tank, drip tank, rinse tank, and waste tank. The robot places the standard sample vial in the sample tank, and then the small robotic arm moves to the sample vial and uses the extraction device to draw the quantitative catalyst ink, which is then transferred and dripped onto the substrate stored in the drip tank. The substrate carrying the sample is then sent to the electrocatalytic workstation for electrochemical performance testing. The small robotic arm that completes the work moves to the rinse tank, extracts anhydrous ethanol to clean the suction line, and finally discharges the cleaning solution into the waste tank. The electrochemical workstation can perform automatic one-click measurement [1], and automatically generate experimental reports using a Python code. To control the effective working area of the carbon paper electrode so that all the conditions for electro-catalytic OER experiments are consistent, the following procedure are applied: First, the carbon paper is cut into rectangle to serve as substrate, and then the working part is isolated separately by hydrophobic epoxy resin with a fixed area of 5 cm^2^. Then, the catalyst ink is coated by the liquid dispensing station, only the working area is covered with catalyst layer while the rest part of carbon paper is blank. Afterwards, the working electrode undergoes a period of natural air drying, the AI-Chemist completes subsequent testing by clamping and transferring it to the electrochemical workstation.

**
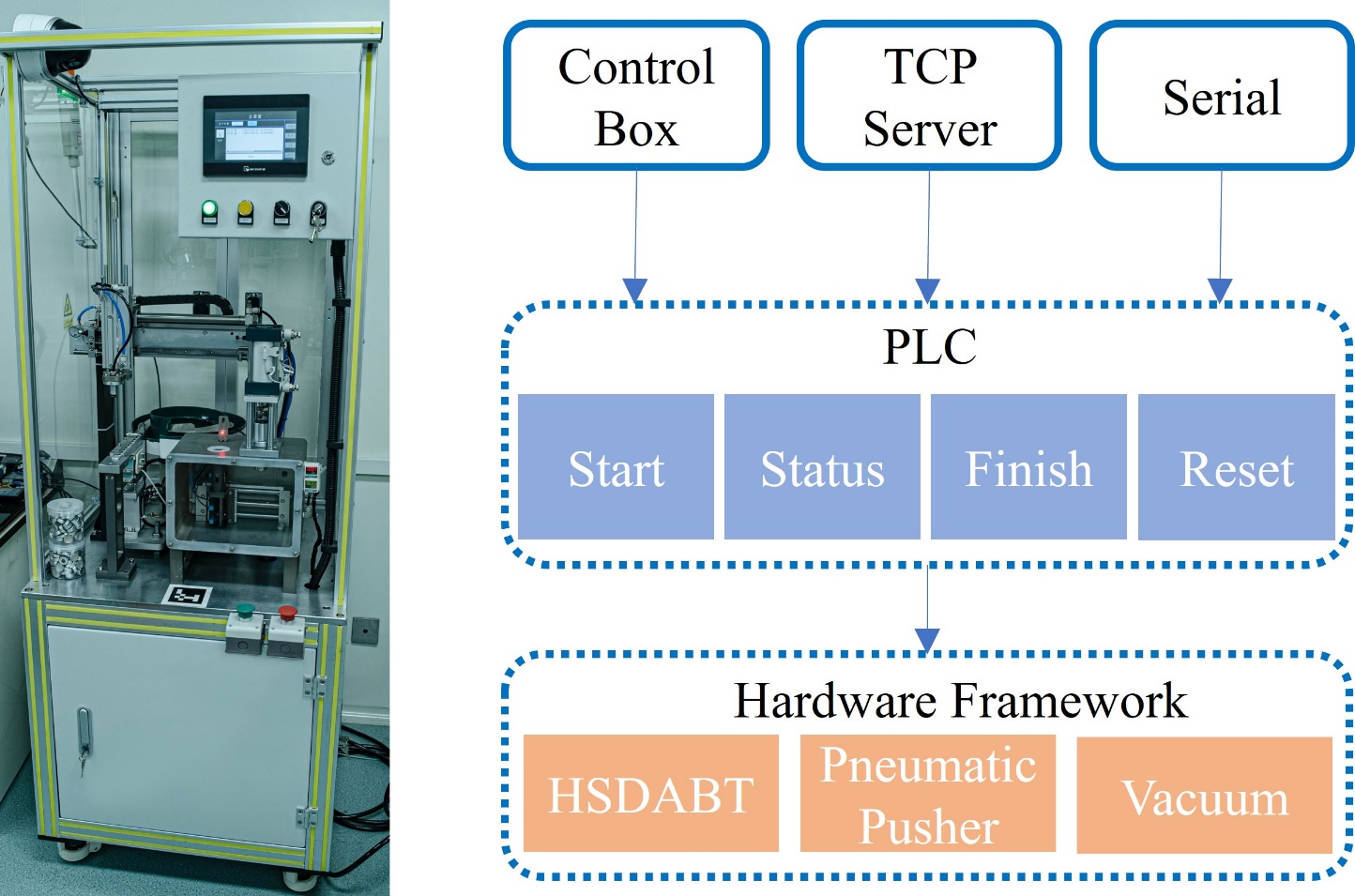
**

**Figure S22.** Capping workstation. The capping workstation is a self-developed workstation used to carry out vacuuming, gas filling and sealing operations for the sample vials. The workstation includes a vibrating plate, linear guides for bottle cap movement, and a sealed chamber for vacuuming and gas replenish (including He, N_2_, Ar, CO_2_, O_2_, H_2_, etc.) to meet different requirements for various experimental tasks. The workstation uses Programmable Logic Controller (PLC) to automatically control the overall workflow, in which the sample vial containing the reactant is first subjected to the capping operation, and then sent to the photocatalytic workstation for the light irradiation.

**
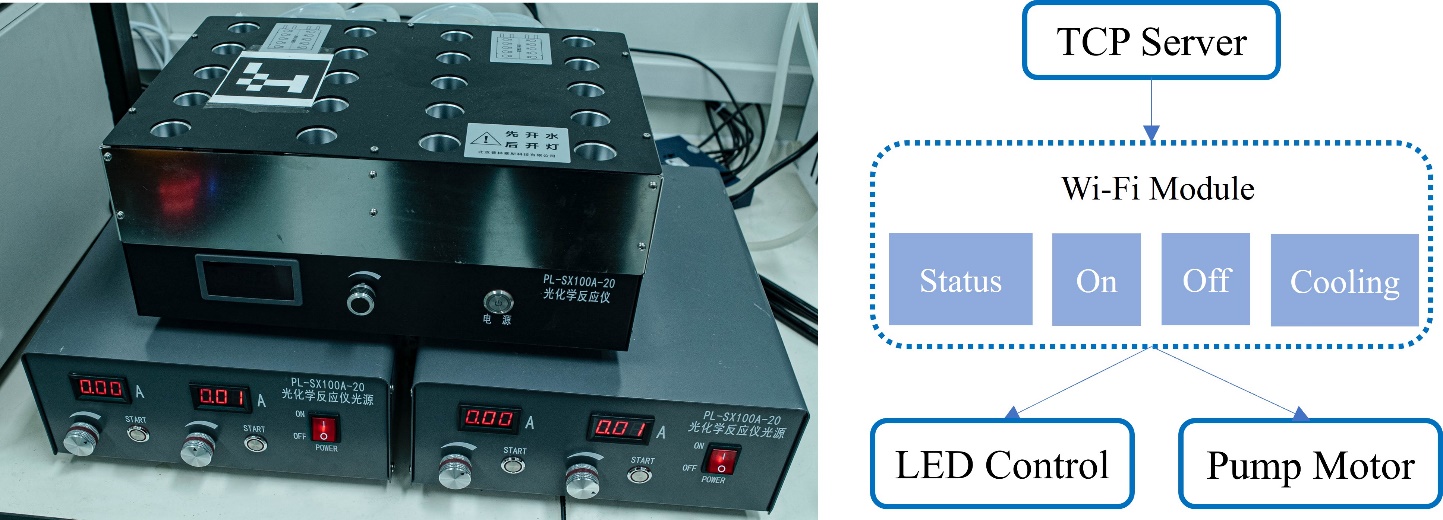
**

**Figure S23.** Photocatalysis workstation. The photocatalysis workstation includes a photoreaction pool, a power supply, and an internal circulation condensation system, the lowest temperature can reach -12 °C. This workstation can simultaneously perform light response tests on 20 standard vials. The device is remotely controlled by adding a wireless communication module (ESP8266) to achieve millisecond-level precise control of the light response time.

**
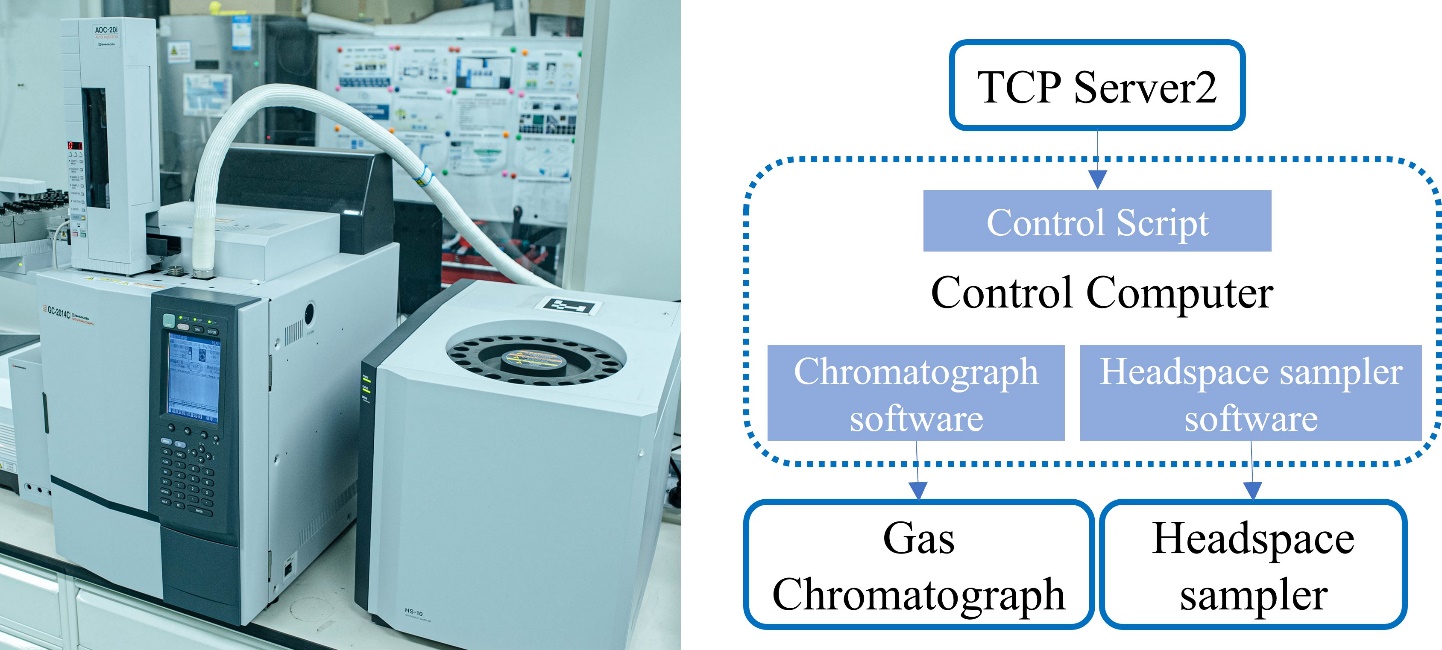
**

**Figure S24.** HS gas chromatography (GC) workstation. The HS gas chromatography (GC) workstation includes a commercial gas chromatography analyzer (Shimadzu GC-2014C), a headspace sampler (Shimadzu HS-10), and a computer. The headspace sampler can continuously inject samples from 20 standard vials to the gas chromatograph analyzer. The supporting instrument software is independently developed. The self-developed software is similar to that for UV/Vis absorption spectroscopy workstation.

# Experimental Section

**Optimization the fluorescence condition of biocompatible aggregation-induced emission (AIE) luminogen**

Berberine molecules (BBR) and analytical grade tetrahydrofuran (THF) were commercially available from Macklin Biochemical Co. Ltd (Shanghai, China). Using the solid dispensing workstation and liquid dispensing workstation, a series of solutions were prepared in a mixture of 40 vol% H_2_O and 60 vol% THF with progressively increased BBR concentrations from 0.5, 5, 10, 15, 20, 25 to 30 mM. Other solutions of BBR with a concentration of 20 mM were also prepared, while varying the volume ratio of H_2_O/(H_2_O+THF) from 10% to 100%. The corresponding PL spectra of these BBR solution were measured in the fluorescence spectroscopy workstation with excitation wavelength of 405 nm.

**Photocatalytic degradation of pollutants and dye-sensitized photocatalytic water splitting**

The steps for the synthesis of hydrogenated materials are as follows: First, 10 mL of dilute hydrochloric acid solution (4 mol/L), commercial MoO_3_ powder (0.1 g) and a certain mass of metal Cu were added to the vial and transferred to the magnetic stirring workstation to initiate the reaction with time duration of 2 h. Subsequently, solid-liquid separation was performed at 4000 r/min for 2 min and the waste liquid was removed from the top of the vial. Deionized water was added to wash the impurity ions adsorbed on the material, and the centrifugation and solid-liquid separation process was repeated as described above. The tunable hydrogenation of MoO_3_ with different concentration of H-dopants was realized by varying the initial Cu mass from 0.005, 0.01, 0.02, 0.04, 0.06, 0.08 to 0.1 g. In the Figure 4E and 4F, they were labeled as HMO-1 to HMO-7, respectively. For the photocatalytic pollutant degradation, the vials containing obtained samples were injected with 10 mL of aqueous solution containing 10^-5^ mol/L Rhodamine B (RhB), which was light irradiated at the photocatalysis workstation under magnetic stirring. The concentration of RhB remaining in the solution at the end of the photodegradation was measured by UV-Vis spectroscopy. While for the dye-sensitized photocatalytic water splitting experiment, the vials containing obtained samples were injected with 10 mL of aqueous solution containing 0.6 mM Eosin-Y and 1.5 M triethanolamine (TEOA). Before irradiation, the vials were vacuum sealed at the capping workstation. The amount of H_2_ produced in the vial after irradiation was quantitatively determined by gas chromatography. The light source for all the photocatalytic reactions was a 25 W white LED lamp and the irradiation time was set to 1 h.

**High-entropy catalyst for electrocatalytic oxygen evolution reaction (OER)**

Metal–organic frameworks (MOFs) containing five metals (Co, Ni, Mn, Cu and Zn) were selected to study the electrocatalytic oxygen evolution performance under alkaline conditions. Acetates of these metals were firstly dissolved in DMF to form the precursor solutions with equimolar concentration of 0.05 mol/L. Different volumes of metal salt solutions were added to adjust the ratio of metals in the final product to meet the definition of high-entropy material (each single metal content ranges from 5% to 35%), and the total volume in the vial was kept at 10 mL after precursor injection. Then, 3 mL DMF solution containing 0.25 mol/L terephthalic acid was added and the mixture was stirred at room temperature and ambient conditions for 1 h. Finally, the solid-liquid separation was performed at 6000 r/min for 2 min and the waste liquid was removed from the top of the vial. The resulting precipitate was washed once with ethanol to remove excess impurity ion. The catalyst ink was prepared by dispersing the as-prepared MOFs in 8 mL of a mixed solution of isopropanol (7.8 mL) and 5 wt% Nafion (0.2 mL) under magnetic stirring. Afterwards, the catalyst ink (350 μL) was dropped onto the surface of carbon paper and naturally dried. All the electrochemical measurements were conducted at the electrochemical workstation (CHI-660E, CH Instruments) in a standard three-electrode system with the high-entropy MOFs as the working electrode, a graphite rod as the counter electrode and Ag/AgCl in saturated KCl as reference electrode. The OER tests were performed in 1 M KOH solution, and the applied potential were calibrated to reversible hydrogen electrode (RHE) following the equation E_RHE_ = E_Ag/AgCl_ + 0.0591×pH + 0.197 V.

According to the tested the XRD patterns of the as-prepared optimal high-entropy OER catalyst (Co_0.35_Ni_0.24_Mn_0.08_Cu_0.28_Zn_0.05_-MOFs) shown in Fig. S47, it does not exhibit any distinct X-ray diffraction peaks. In comparison with the most common structures of MOFs (MIL-101, MOF-74, MOF-71) usually formed from metals and terephthalic acid used in our experiments, we found that the diffraction intensity of our high-entropy MOFs material was so weak as to obtaining the clear crystal structure from diffraction data becomes almost impossible. This is because the greater the enthalpy and entropy values of a particle, the lower its crystallinity. The increased high-entropy effects and multiple doped metal atoms with different radius in high-entropy material usually promote the formation of low crystallinity to accommodate lattice distortions and release corresponding complex stress mutations. In addition, we calibrated the catalytic activity sites of metal-oxygen cluster in the MOFs structures using MIL-101, MOF-74 and MOF-71 based on the experimental results from AI-Chemist and found that only MIL-101 matched our theoretical prediction of the expected catalytic activity, i.e., the OER overpotential of single-component MOFs follows the order Zn < Cu < Mn < Ni < Co (Fig. S48). Therefore, based on the above considerations, we chose MIL-101 as the model structure for theoretical simulations.

# Proposing Scientific Hypothesis

NLP is the first step in the automated design of the experimental protocol in this work. It mines data from a large number of relevant literatures, analyzes these data statistically, and gives a general direction of the experiment based on the statistical results. We designed an automated "search-mining-analysis" process that can help robots to quickly understand the development and characteristics of a specific field. We used some machine learning models (e.g., tokenization, named entity recognition, etc.) and some rules (e.g., grammar rules, compound naming rules) to guarantee the accuracy of the data. NLP models generally face accuracy problems, so we do not rely too heavily on the data mined by NLP. After getting the data, we also analyzed the data statistically and adopted the most probable part of the statistical results, which can filter out a lot of erroneous data. On the other hand, the NLP is only the first step in the automated design of experimental protocols, which only gives the general direction of the experimental protocols, more precise guidance of the experimental protocol relies on subsequent theoretical calculations, machine learning, and automated exploration of the robot, which also reduces misleading experiments from erroneous data. The details of NLP are described below: Firstly, we automatically searching for abstracts or full text of relevant papers based on keywords in the area of interest. Next, we used NLP tools such as ChemDataExtractor and OSCAR4 to mine the chemical entities in the literature, the nature of the entities, and the relationships between the entities. Then, statistical analysis of the data mined by NLP was performed to find general directions for further research. The most probable part of the statistical results was adopted to avoid the effect of erroneous data. The NLP gives the general direction of the experimental protocols, while more precise guidance of the experimental protocol relies on subsequent theoretical calculations, machine learning, and automated exploration of the robot, which also reduces misleading from erroneous data.

# Navigation and Planning

**Figure S25.** Laboratory map and workstation distribution. The robot uses the Simultaneous Localization and Mapping (SLAM) algorithm to obtain its localization information and laboratory map for the navigation and planning module. The point cloud information of two HOKUYO lidars is fused, which can detect the surrounding environment information in 360° directions. We use the graph-optimized Karto SLAM algorithm to rapidly construct 2D grid maps of the laboratory. In addition, based on the established maps, an Adaptive Monte Carlo Localization (AMCL) algorithm is used to fuse the odometry information of the omnidirectional mobile platform to obtain the current localization of the robot. During the experiment, combined the current global pose of the robot with the position of the target workstation, a two-stage planning method was designed to achieve smooth motion between different experimental workstations. The first stage is the path planning from the current position of the robot to the target workstation so that the robot can reach the target workstation for experimental operations. The second stage is to design an S-shaped speed planning based on the kinematic model of the omnidirectional mobile platform to ensure the stability of the robot’s motion. At the same time, the surrounding information of obstacles will be detected in real-time according to the data of the lidar, which realizes autonomous and safe obstacle avoidance.

# Perception and Measurement

## Vision-Based Target Recognition and Pose Measurement

The mobile robot uses the Intel RealSense D435i depth camera, including two imagers, an infrared projector, and an RGB camera module. The output resolution of the depth image is 1280×720, and the resolution of the RGB camera is 1920×1080. Considering that the localization accuracy of the robotic arm gripper should be high in the experimental operation, the ArUco label [2] was used to construct a structured laboratory scene. For the 14 experimental workstations, an ArUco label with an independent ID was assigned to the instrument in each workstation. Fig. S26 shows the ArUco label assigned to the centrifuge with an ID of 38. We predefined an ArUco label dictionary containing all labels assigned to the instruments of experimental workstations. Binarization, contour extraction, and filtering are performed on the images collected by the camera to obtain the outer contours of the candidate square labels. The local regions within these contours are template-matched with the dictionary, and the label with the most similar features is searched. Thereby we can obtain the label ID and its contour on the current image, and then calculate the label pose through the PnP algorithm. After pre-calibrating the relative pose between the target to be operated (including instrument buttons, sample vials, sample racks, etc.) and the fixed label, the robotic arm can detect the pose of the target to be operated by measuring the pose of the label during work. In this way, experimental operations such as pressing buttons, grasping sample vials, and grasping sample racks are completed. After assigning an independent ArUco label to the instrument in each experimental workstation, one can fix the label on the instrument and keep it relatively stationary with the instrument. The robot judges the current workstation by visually identifying the label ID in the field of view, and realizes the accurate measurement of the workstation pose based on vision according to the relative pose between the ArUco label and the target to be operated in the experimental workstation. Because the label and the instrument are relatively fixed, even if the instrument moves under unknown factors, the robotic arm can accurately locate the target and complete the operation task.


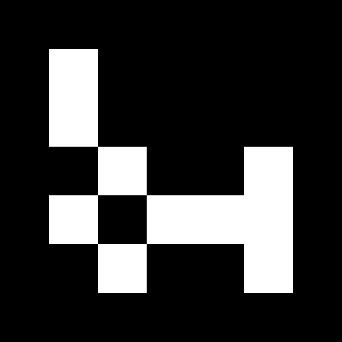


**Figure S26.** Vision-based target recognition and pose measurement via ArUco-38 label.

## Vision-Based Pose Detection of Centrifuge Holes

During the solution separation operation, the mobile robot is required to independently complete the operation of accurately placing the sample vials into the centrifuge holes and taking them out. Since the position of the rotor is not fixed after centrifugation, to achieve fully autonomous operation, it is first necessary to accurately measure the poses of the rotor and its holes. To this end, we developed a general vision-based pose detection method for the centrifuge rotor and its holes.

As shown in Fig. S27, a measuring plate is fixed on the rotor of the centrifuge. The schematic diagram of the measuring plate is shown in Fig. S28. The measuring plate and the upper surface of the rotor can be completely fitted, and three through-holes are left so that the vials can be put into the holes in the rotor. The ArUco labels are fixed in the three grooves on the measuring plate. By detecting the three ArUco labels, the center pixel coordinates of labels can be obtained, and the average value of these coordinates is calculated to obtain the center pixel of the measuring plate. As shown in Fig. S29, it is assumed that the geometric centers of the three labels are on the XOY plane. The origin is located at the center of the equilateral triangle, and the X-axis passes through the vertex of one of the labels. By mapping the 2D pixel coordinates to the 3D space coordinates one-to-one, the Perspective-n-Point (PnP) problem is established, and the pose of the measuring plate can be obtained by solving this problem. Since the relative pose relationship between the measuring plate and the rotor is fixed, we can get the pose of the central axis coordinate of the rotor. It is the same with the pose of the measuring plate. Then teach the robotic arm to record the relative pose between its end and the rotor’s central axis when the robotic arm grasps and puts a vial into a hole on the rotor completely, and obtain the accurate measurement result that satisfies the requirement of putting the vials into the holes by the robotic arm.

As shown in Fig. S30, we pre-recorded the relative pose relationship between the end of the robotic arm and the rotor when the gripper puts the vials into each centrifugal hole. During the practical working process, the mobile robot arrived near the centrifuge. After measuring the current pose of the centrifuge rotor, the target pose of the end of the robotic arm was calculated according to the relative pose relationship pre-recorded.


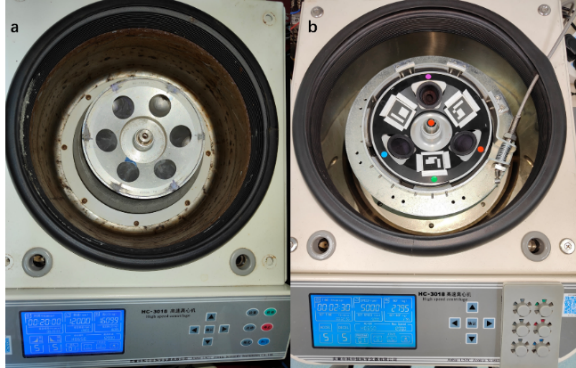


**Figure S27.** Vision-based pose detection of centrifuge holes. (a) Original centrifuge and (b) Centrifuge with measuring plate installed.


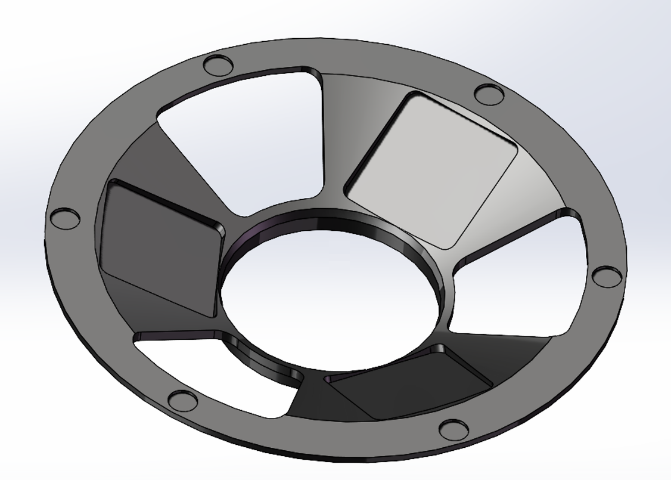


**Figure S28.** Schematic diagram of the structure of the measuring plate.


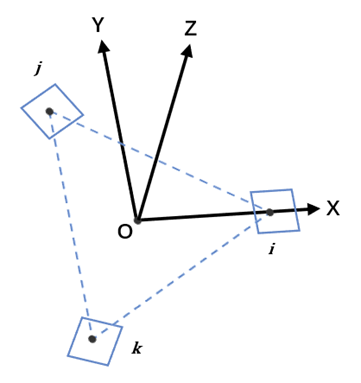


**Figure S29.** Schematic diagram of the coordinate system of the centrifuge measuring plate. Here points *i*, *j*, *k* represent the geometric centers of the three labels.


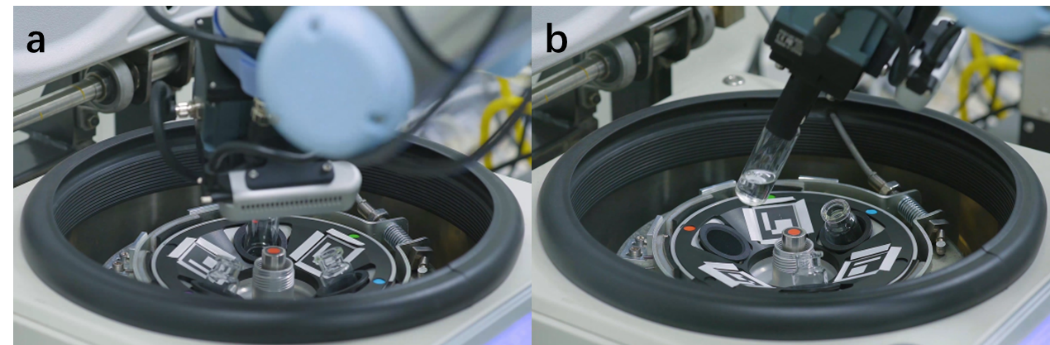


**Figure S30.** Precise localization and operation of the centrifuge holes. (a) Put the sample vial into the hole. (b) Take out the sample vial from the hole.

## Vial Detection and Localization Method Based on Deep Learning

There are some abnormal situations during the experimental operation, such as an empty grasp of the gripper, sample vial falling from the gripper, missing some sample vials on the vial rack, etc. Therefore, we need to detect and locate transparent vials in unstructured scenarios, to handle these situations and ensure the normal running of the fully autonomous experiment process.

Because infrared light will transmit when irradiating transparent objects, it is difficult for an infrared depth camera to accurately measure the depth of transparent objects, resulting in the lack of depth image information. It is a challenging task to realize the detection and localization of transparent vials in unstructured scenes. Sajjan *et al*. proposed a scheme named ClearGrasp based on deep learning [3] which fused three neural networks including normal estimation, boundary detection, and transparent object segmentation to detect transparent objects in color images and their attribute. Then the attribute was used for depth completion of images with lacked depth information to obtain a complete depth image. During the experimental operation, robotic arm is required to grasp the top mouth of a sample vial, so that the mouth can be kept upward to prevent the internal liquid from spilling. Therefore, only the depth image is insufficient to c complete this task, and it is necessary to further locate the position of the mouth. Liu *et al*. proposed a multi-view 3D labeling method named KeyPose based on deep learning [4] to predict the key point pixels of transparent objects from color images. Based on the above two works, we develop a method to detect and locate transparent vials in unstructured scenarios, and the process is shown in Fig. S31. The specific implementation process is as follows:

1) D435i depth camera is used to collect the registered color image and depth image as inputs, here the depth image is lacking information;

2) Process the color image and the depth image with lacking information through ClearGrasp, and output the binary mask map of the transparent objects and the completed depth image;

3) Perform the minimum rectangle contour fitting on each mask in the binary mask map, calculate the upper and lower midpoints of the rectangle, and input them as pseudo key points of KeyPose;

4) Process the color image and pseudo key points through KeyPose, and output the predicted real key point pixel coordinates. Since the color image and the depth image are completely registered, the color image key point pixel coordinates can be directly used as the pixel coordinates of the depth image;

5) Since each pixel value on the depth image is a depth value, the corresponding depth value can be obtained according to the pixel coordinates of the key points in the previous step, and then the corresponding 3D-space coordinates can be calculated using the SDK inside the camera. These 3D-space coordinates are the positions of the mouths on the sample vials used for grasping by the robotic arm.

Through the above steps, we have realized the detection and localization of transparent vials, as shown in Fig. S32, here the blue points in (a) and (c) are the predicted key points of the vials, and (b) and (d) are the completed depth images. As can be seen, the predicted key points are correct. This method can be used to detect and locate the sample vials in real time when there are abnormal situations, thereby greatly improving the accuracy and reliability of experimental operations.


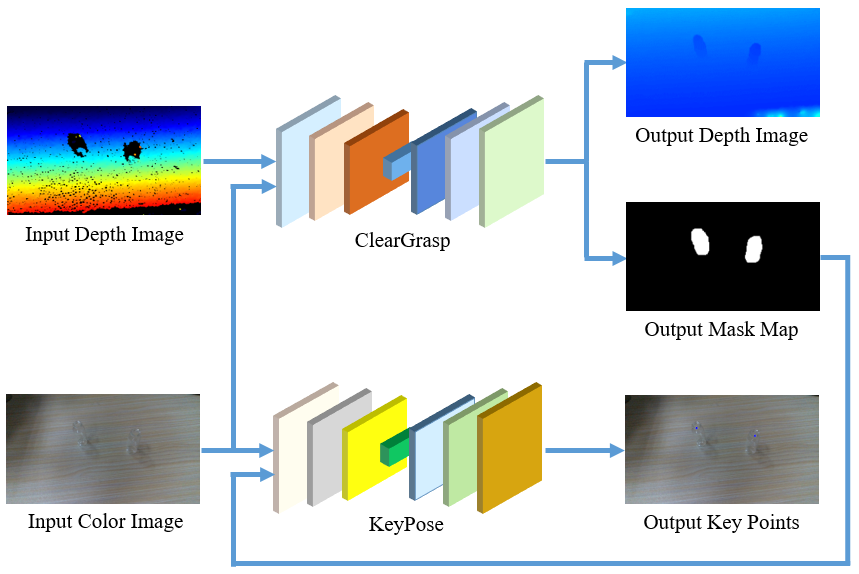


**Figure S31.** Schematic diagram of the detection and positioning process for transparent sample vials.


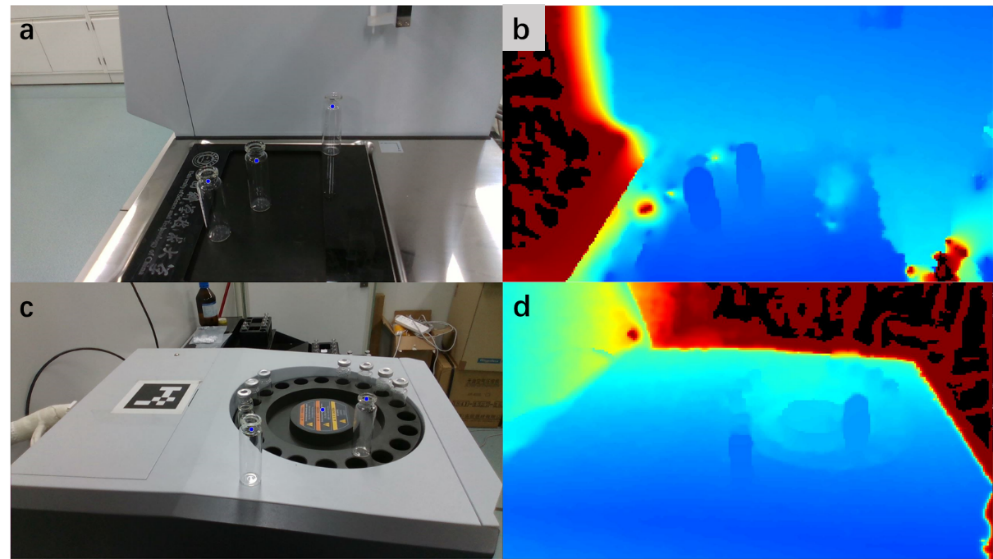


**Figure S32.** Experiment result of detection and localization for transparent vials in a complex environment. The blue points in (a) and (c) are the key points predicted by the model, (b) and (d) are the corresponding completed depth images.

# Task Management of The Robot

The task management module manages and makes decisions for the movement and operation of the robot using the finite state machine, and plays the same role as the "brain" in the whole robot system. Its main functions include: 1) communicating with the workflow management module to receive experimental workstation tasks and detect the robot status; 2) communicating with the navigation and planning module to issue movement instructions of the mobile platform and receive feedback of movement results; communicating with the grasping operation module to issue robotic arm operation instructions and receive feedback of operation results; 3) communicating with each chemical instrument in LAN to control the instrument to start or stop working.

For the movement and operation scenarios of the robot in each experimental workstation, the task management module designs a main state machine main-FSM = (Q, Σ, δ, q0, F), and its directed graph is shown in Fig. S33. The set of States Q = {S0, S1, … , S7}. , here S0 represents the initial state, that is, the experimental workstation task is received; S1 represents the state "ensure that the robotic arm is reset before moving"; S2 represents the state "move to the target workstation"; S3 represents the state "relocation of workstation"; S4 represents the state "in a certain sub-state machine". Specifically, according to the different experimental operations of all 14 workstations in the scene, 14 workstation sub-state machines are designed here sub-FSM = {S4-1, S4-2, … , S4-14}; where S5 represents the state "complete all movements and operations of the workstation"; S6 represents the state "abnormal"; S7 represents the termination state, that is, complete the workstation instruction and feedback. In addition, Σ is the input alphabet, that is, the input of each state, Σ = { the corresponding movement or operation task is completed, an exception occurred and the task cannot be completed }; δ represents the state transition function δ: Q × Σ → Q, and the jump relationship between each state in the directed graph of the state machine is the formal representation of the state transition function; q0 represents the start state of main-FSM, q0 = S0; F represents the set of termination states of the main-FSM, F = {S7}.

The sub-state machine sub-FSM, part S4 in Fig. S33, is designed for 14 workstations for different robotic experimental operations and different chemical instrument communications involved in different workstations. Taking the centrifugal workstation as an example, the directed graph of the S4-6 sub-state machine "centrifugal workstation" is given below, as shown in Fig. S34. After the end of the state S3, enter the "input" or "output" state according to the received experimental task instruction of "putting in the centrifugal task" or "taking back the centrifugal task". If the corresponding operation task is completed, the state jumps down in turn until the state S5, and if the operation is abnormal, it jumps to the "abnormal" state S6. In addition, the orange box in Fig. S34 represents the LAN communication status between the robot and the centrifuge, and ‘curr_bottle’ represents the current sample bottle number, which is 1, 2, and 3 respectively.

In addition, considering the abnormal situations in the operation of the chemical experiment, the state machine designs the "abnormal" state S6, which is to solve the problems that occur during the execution of the entire state machine from the state S1 to the states S2, S3, S4 until the state S5. When an abnormal situation occurs, the robot system also can be stable and reliable by stopping the robotic arm in time and recovering from the abnormal situation. Specifically, S6 includes 6 different abnormal sub-states: the camera fails to detect the ArUco tag, the gripper grasps empty, the object falls during the grasping process, the trajectory planning of the robotic arm is wrong, the robotic arm collides and stops, and the error occurs again during the abnormal recovery. When the state machine jumps to S6, the robot will no longer perform the task of the current workstation but stop in time, and then jump to S7 to feedback to the workflow management module, and perform abnormal recovery processing according to the abnormal sub-state.

**Figure S33.** The directed graph of the main state machine.

**Figure S34.** The directed graph of the S4-6 sub-state machine "centrifugal workstation".

# Smart Chemical System

For the dye-sensitized photocatalytic water splitting experiment, we assume that 8 groups of MoO_3_ samples (The initial feeding mass is 0.1g.) with different hydrogen doping content need to be prepared, and the 8 experimental groups were reacted with different metal Cu with mass ranging from 0, 0.005, 0.01, 0.02, 0.04, 0.06, 0.08 to 0.1 g in dilute hydrochloric acid solution. Each group of experiments was repeated quintuple times, and a total of 5*8=40 samples were obtained, which were placed with 4 sample racks. The time spent in each operation and moving step is fixed. The experimental process scheduling results of the robot with and without the multi-task dynamic optimization are shown in Fig. 4G. In Fig. 4G, the vertical axis represents the workstation during the experimental process, the horizontal axis represents the time, and one minimum unit represents the time of 150s. In top diagram of Fig. 4G, the blue lines 1 and 2 represent the test process of No.1 and No.2 sample racks respectively. Since they are not optimized, the blue lines in the overall experimental flow chart will be repeated 4 times, representing the test process of 4 groups of sample racks in turn. The light blue lines represent the movement and operation time of the robot during the test, the dark blue line represents the autonomous running time of the workstation during the test, and the red line represents the waiting time of the robot. In bottom diagram of Fig. 4G, the blue line 1, the green line 2, the yellow line 3 and the orange line 4 respectively represent the test processes of No.1, No.2, No.3, and No.4 sample racks that can be carried by the robot at the same time. The light (blue, green, yellow, and orange) colored lines represent the movement and operation time of the robot during the test, and the dark (blue, green, yellow, and orange) colored lines represent the autonomous running time of the workstation during the test, and the red line represents the waiting time of the robot.

After calculation, it is found that the total time consumption of the 4 sample racks of experiments without optimization is 452.5 * 4 = 1810 minutes, while the total time consumption of the 4 sample racks of experiments after multi-task dynamic optimization is 980 minutes. It can be seen that the multi-task dynamic optimization can significantly shorten the total experiment time and the waiting time of the robot. In addition, if the number of robots and the number of experimental workstations is elevated, the dynamic optimization scheduling of multi-task parallel can bring a more significant improvement effect.

For the electrocatalytic oxygen evolution reaction experiment, we assume that 4 groups of samples with different metal ratios need to be prepared, and the time spent in each operation and movement step is fixed. The experimental process scheduling results of the robot with and without the multi-task dynamic optimization is shown in Fig. 4H. In Fig. 4H, the vertical axis represents the workstation during the experimental process, the horizontal axis represents the time, and one minimum unit represents the time of 150s. In top diagram of Fig. 4H, the blue lines 1 and 2 represent the test process of No.1 and No.2 sample racks respectively. Since they are not optimized, the blue lines in the overall experimental flow chart will be repeated 4 times discretely, representing the test process of 4 groups of sample racks in turn. In bottom diagram of Fig. 4H, the blue line 1, the green line 2, the yellow line 3 and the orange line 4 respectively represent the test processes of No.1, No.2, No.3 and No.4 sample racks that can be carried by the robot at the same time. The light-colored lines represent the movement and operation time of the robot during the test, the dark-colored lines represent the autonomous running time of the workstation during the operation, and the red lines represents the waiting time of the robot.

After calculation, it is found that the total time consumption of the four groups of experiments without optimization is 157.5 * 4 = 630 minutes, while the total time consumption of the four groups of experiments after multi-task dynamic optimization is 365 minutes. It can be seen that the multi-task dynamic optimization can significantly shorten the total experiment time and the waiting time of the robot. In addition, if the number of robots and the number of experimental workstations is elevated, the dynamic optimization scheduling of multi-task parallel can bring a more significant improvement effect.

# Supplementary Experimental Results

**
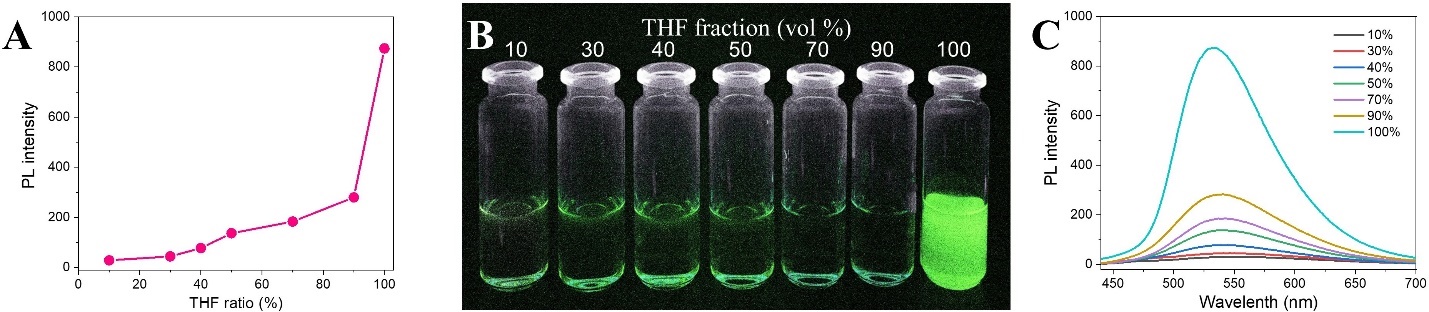
**

**Figure S35.** Relationship between AIE luminescence and THF concentration. (A) Peak fluorescence intensity with different THF fractions; (B) Fluorescence images of the solution in (A) under UV light (365nm); (C) PL spectra of BBR chloride in water and water/THF mixtures with different THF fractions. Excitation wavelength: 405nm.

**
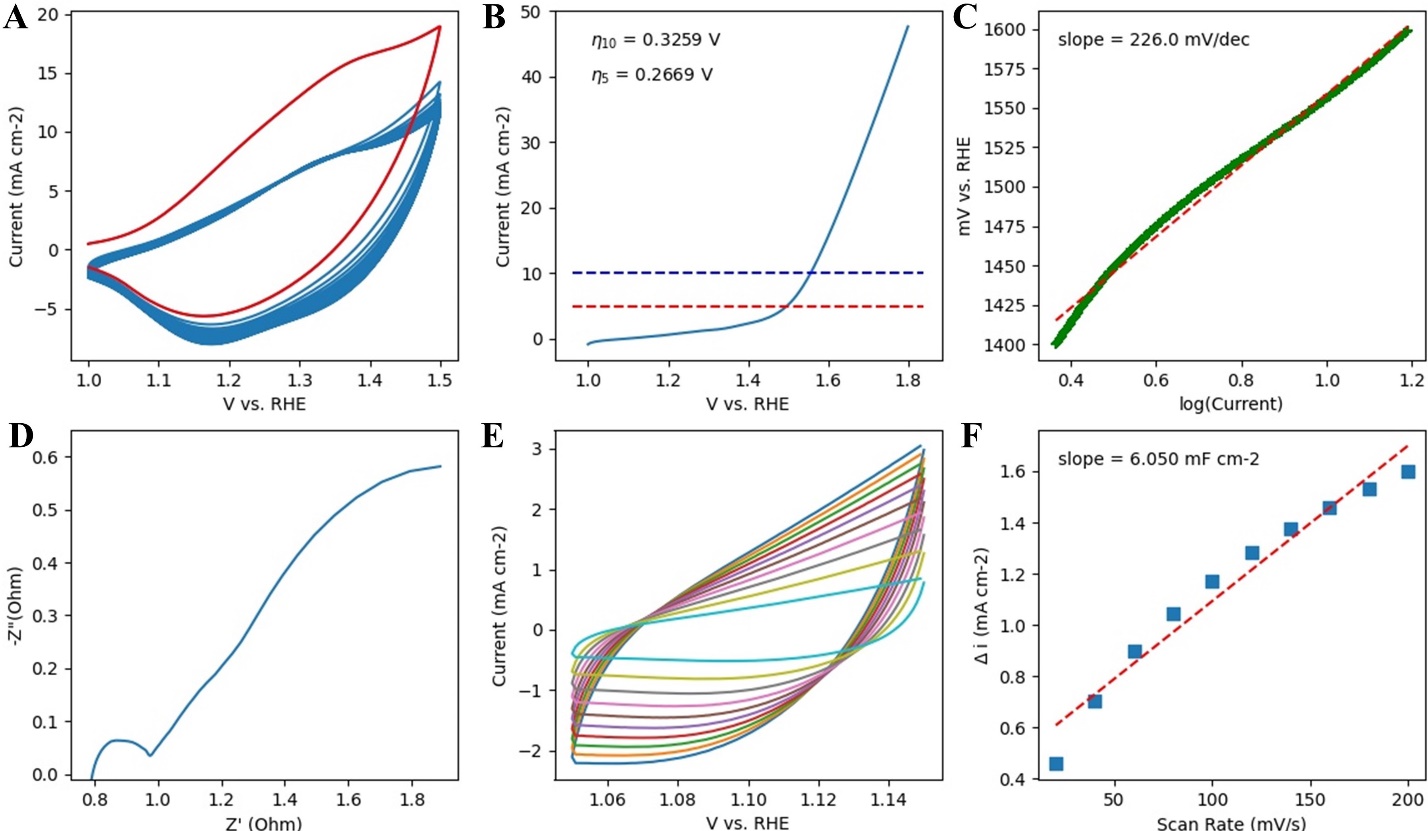
**

**Figure S36.** The experiment report of sample No. 149. (A) Cyclic voltammetry curves (B) Polarization curves (C) Tafel curve (D) Electrochemical impedance spectroscopy (E) Electrochemical C-V curve (F) Electrochemical active surface area.

**
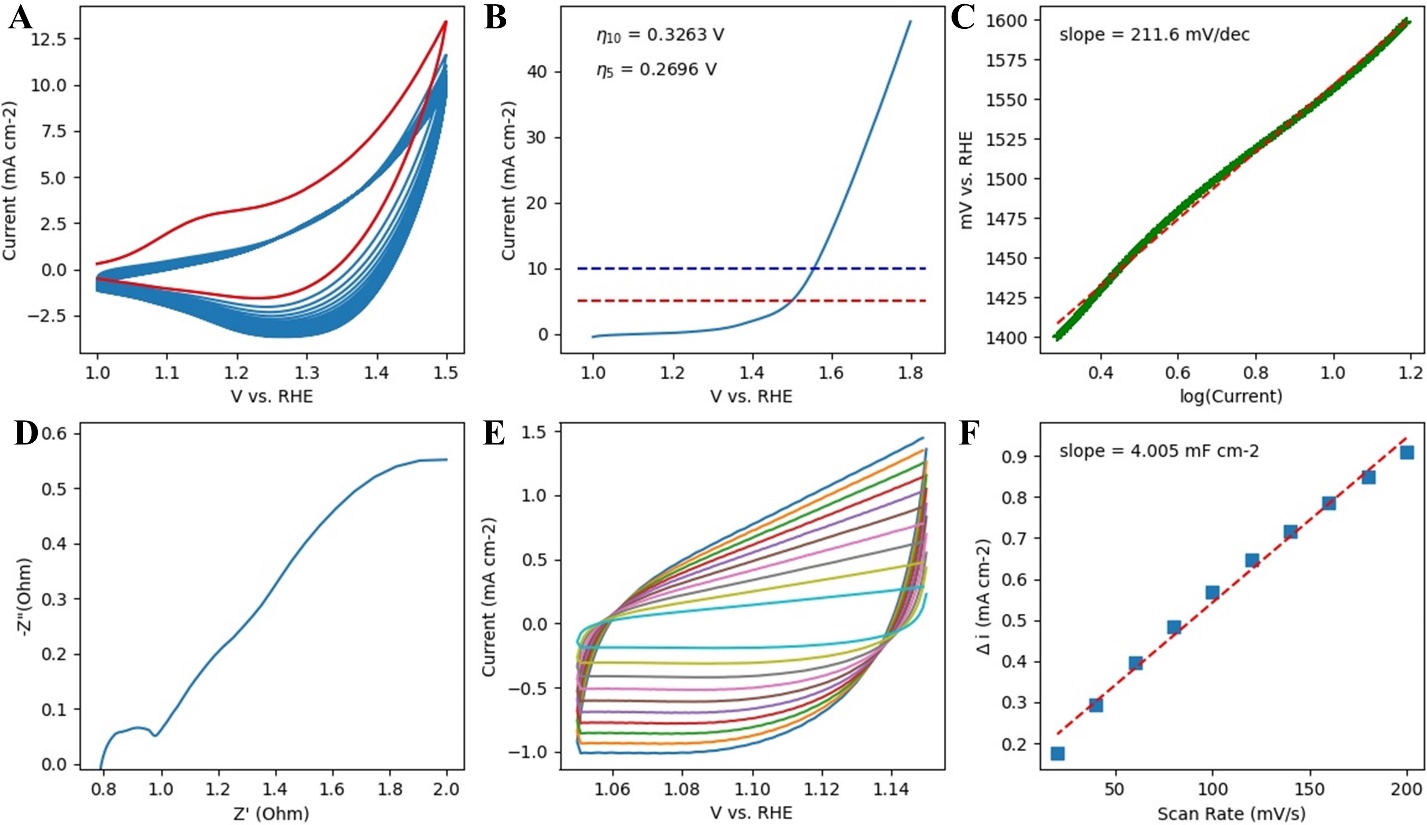
**

**Figure S37.** The experiment report of sample No. 155. (A) Cyclic voltammetry curves (B) Polarization curves (C) Tafel curve (D) Electrochemical impedance spectroscopy (E) Electrochemical C-V curve (F) Electrochemical active surface area.

**
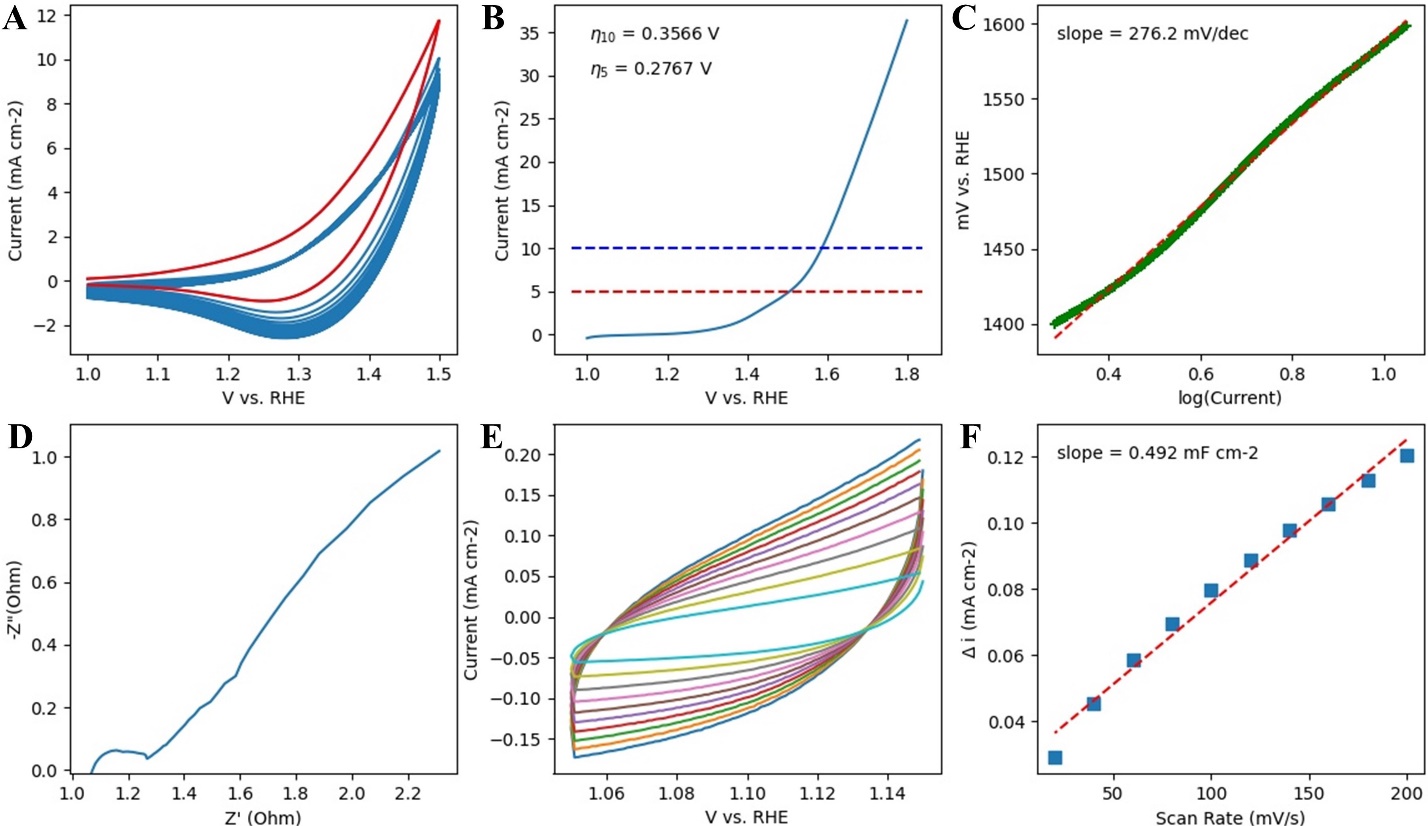
**

**Figure S38.** The experiment report of first optimal sample suggested by Bayesian optimizations based on the experimental data. (A) Cyclic voltammetry curves (B) Polarization curves (C) Tafel curve (D) Electrochemical impedance spectroscopy (E) Electrochemical C-V curve (F) Electrochemical active surface area.

**
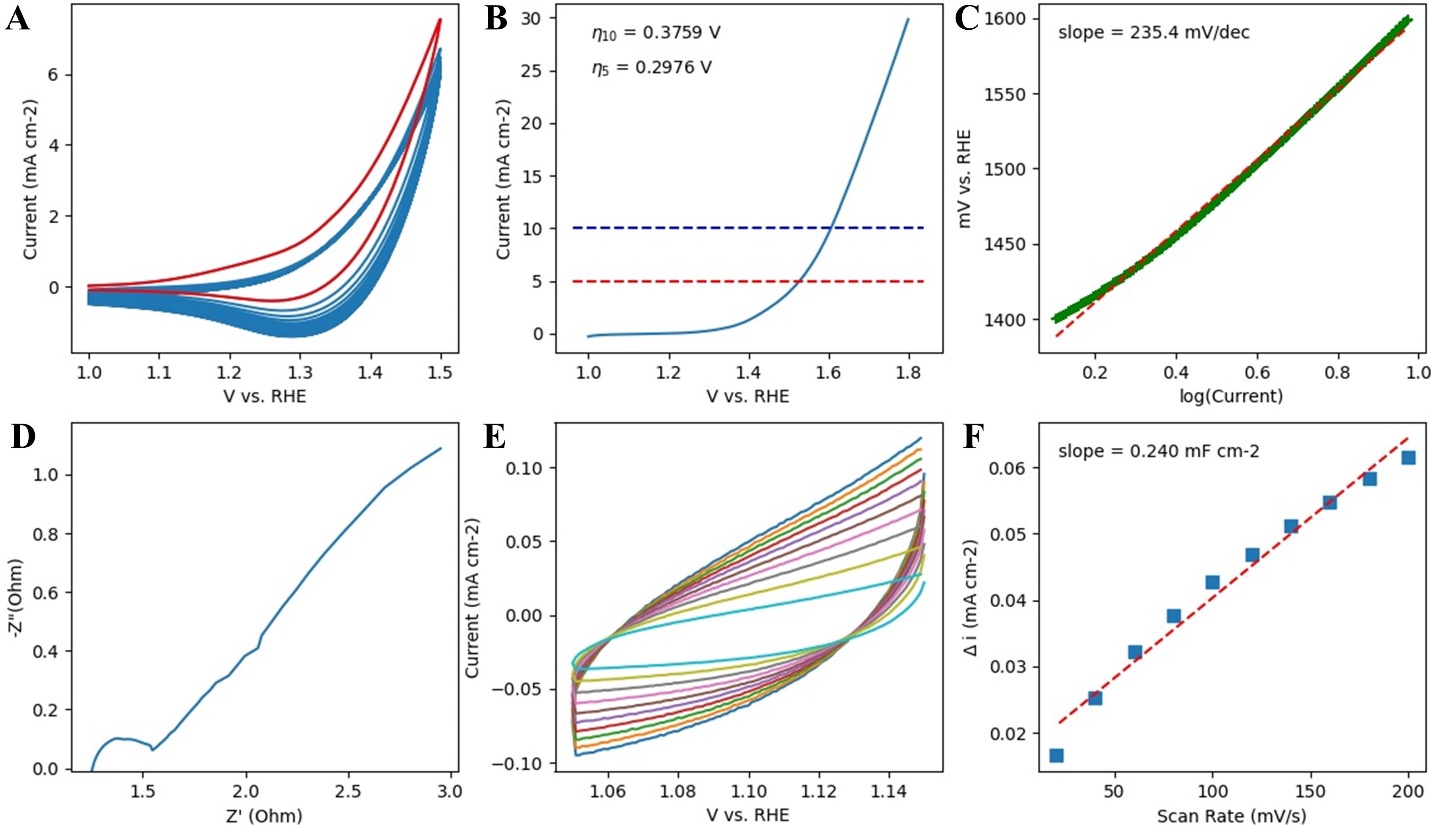
**

**Figure S39.** The experiment report of second optimal sample suggested by Bayesian optimizations based on the experimental data. (A) Cyclic voltammetry curves (B) Polarization curves (C) Tafel curve (D) Electrochemical impedance spectroscopy (E) Electrochemical C-V curve (F) Electrochemical active surface area.

**
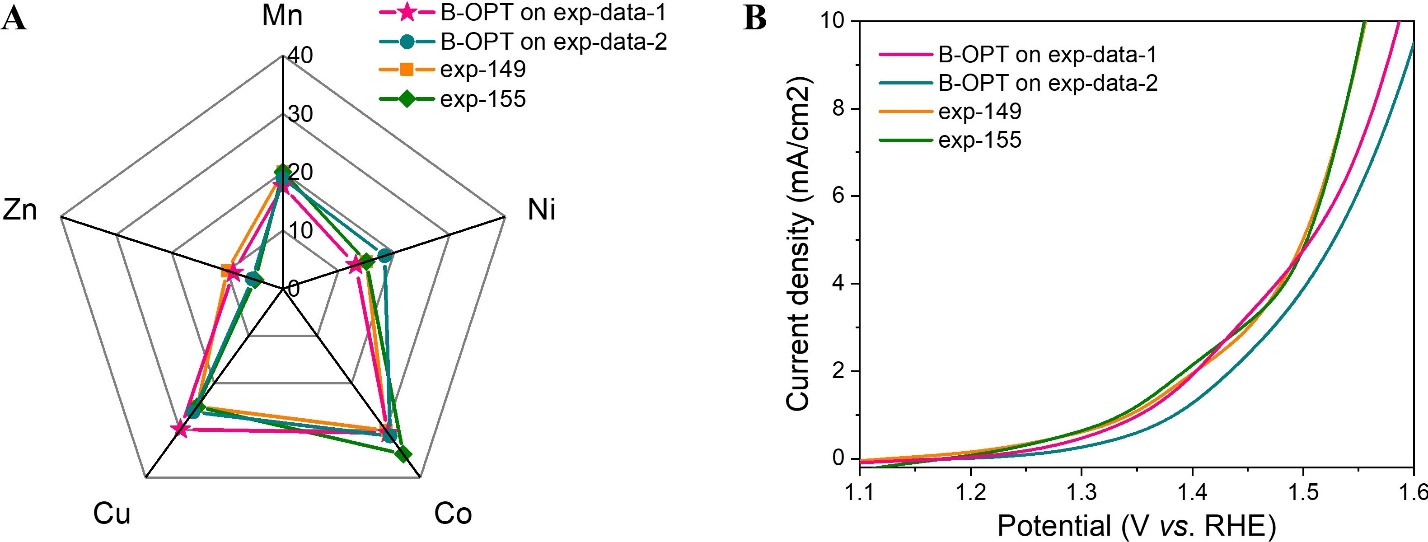
**

**Figure S40.** The comparation between two experimental samples and two optimal samples suggested by Bayesian optimizations based on the experimental data. (A) Kiviat diagram of composition ratios (B) Polarization curves.

**
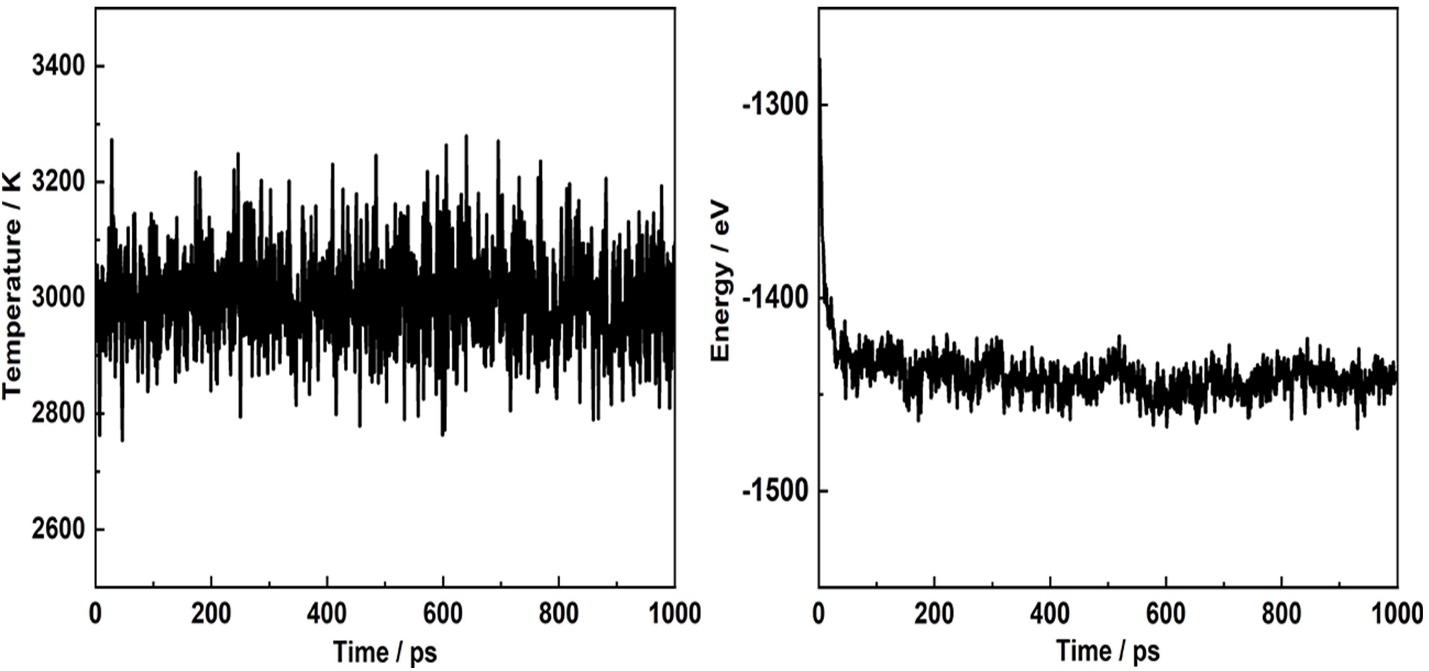
**

**Figure S41.** Temperature and energy versus time curves for the MD simulation.

**
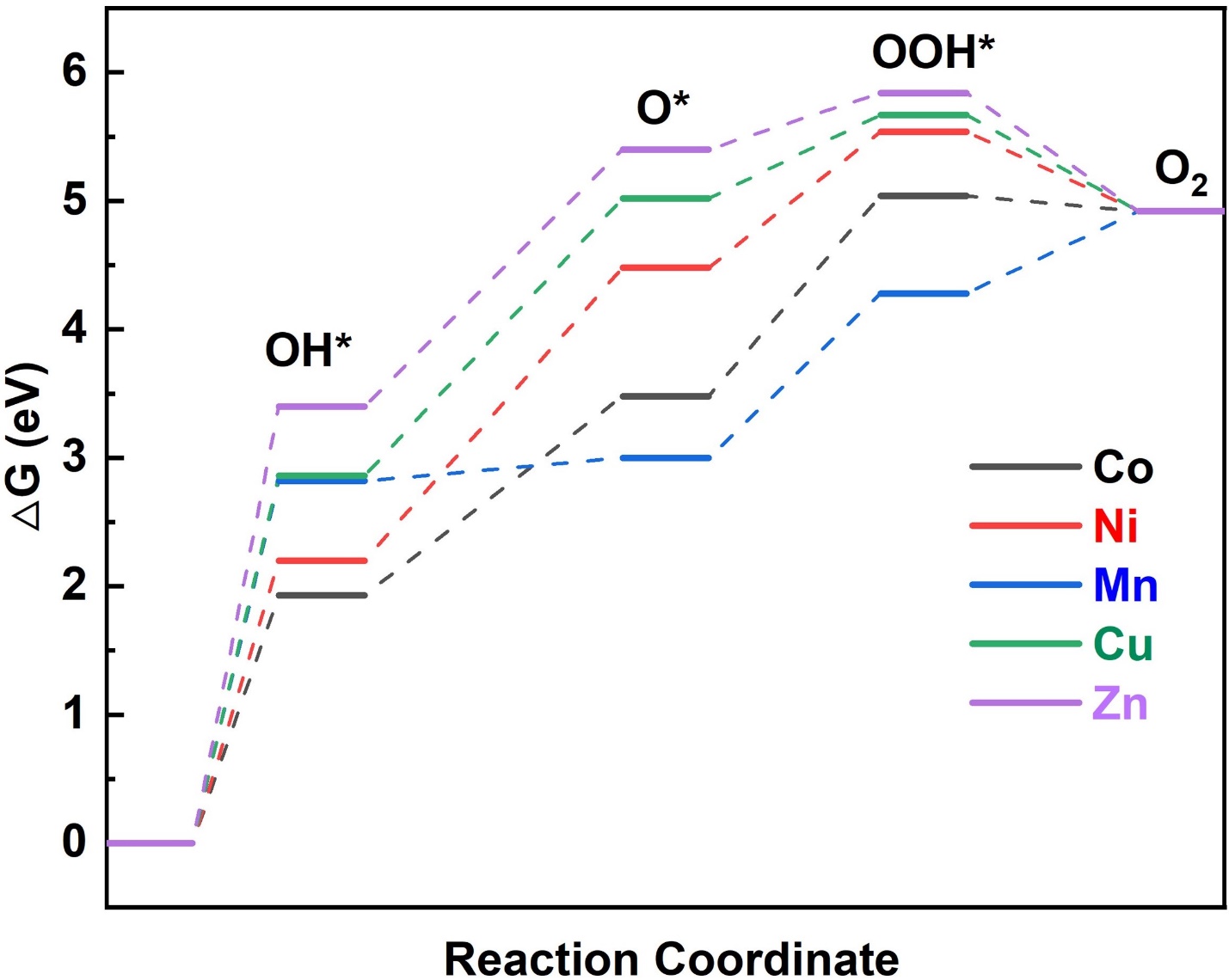
**

**Figure S42.** Free-energy profiles of OER process for MOF clusters of five different metals.

**
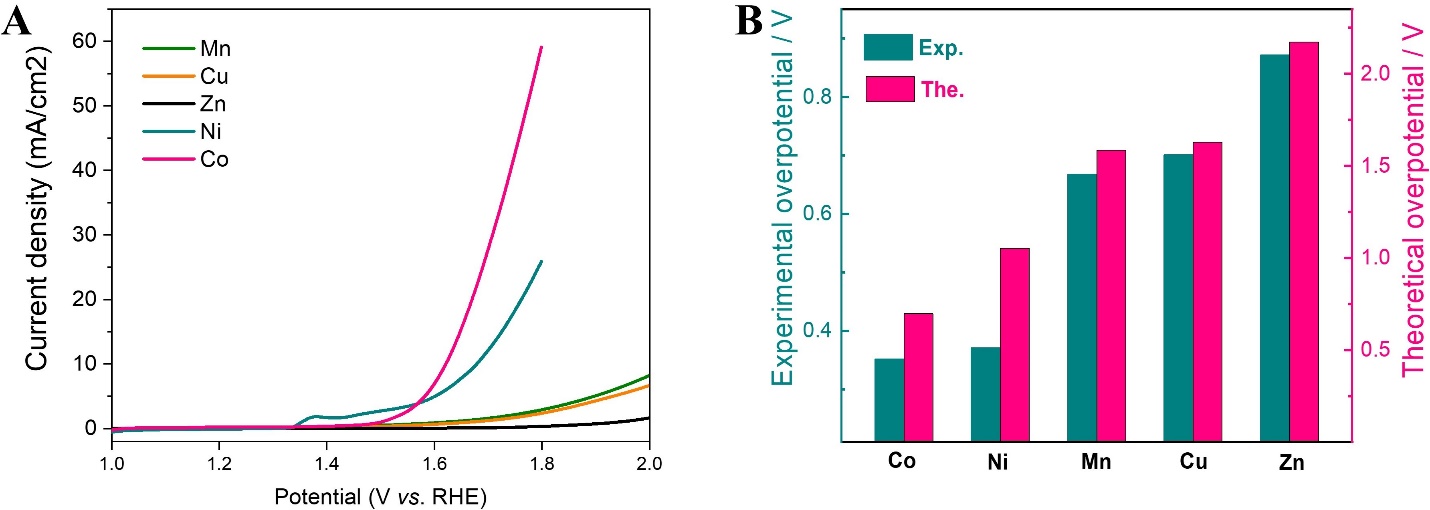
**

**Figure S43.** (A) The polarization curves of MOFs with five different metals. (B) The comparation of overpotentials between experiment and theory.

**
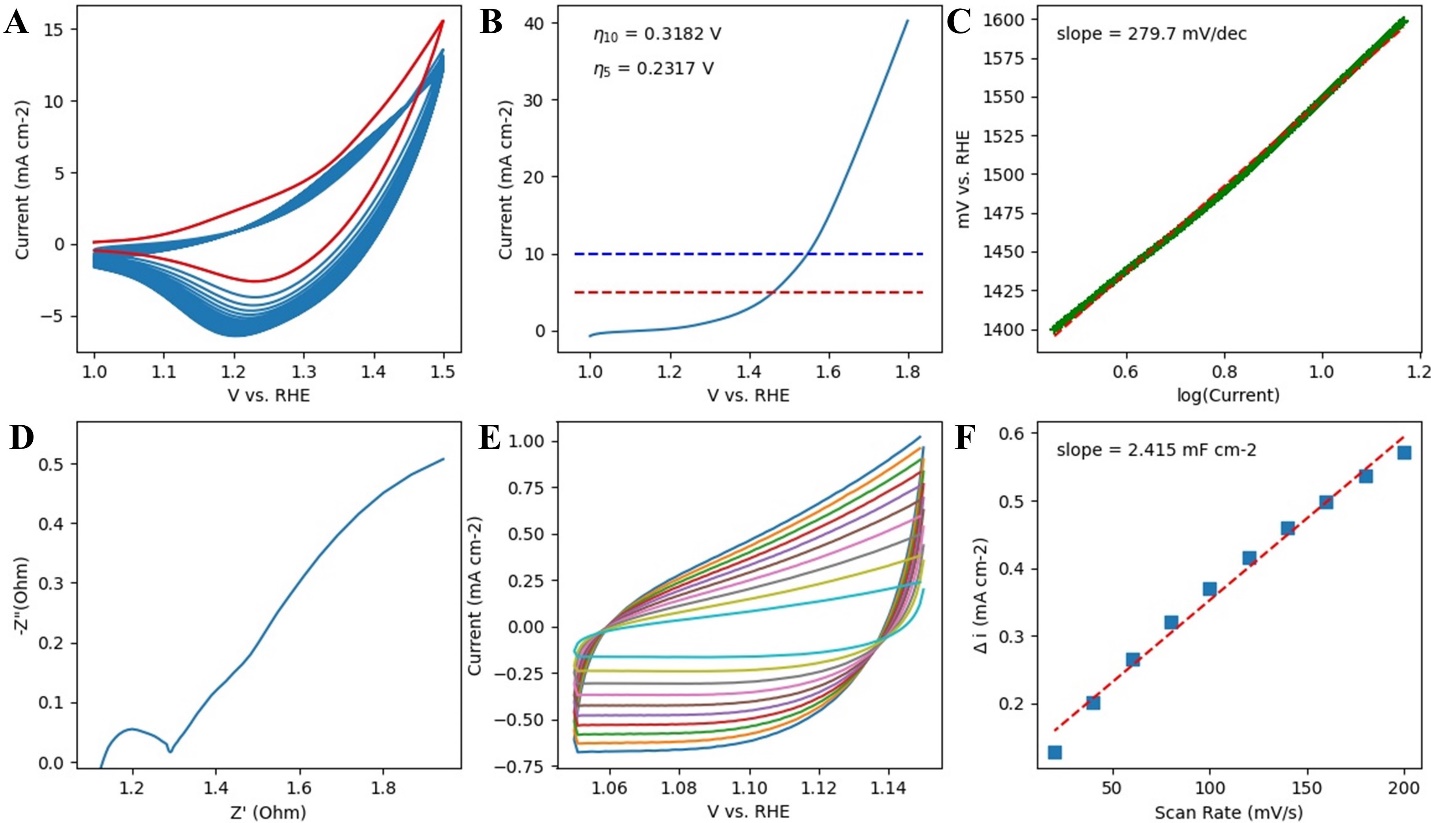
**

**Figure S44.** The experiment report of optimal sample suggested by Bayesian optimizations based on the combination of simulated and experimental datasets. (A) Cyclic voltammetry curves (B) Polarization curves (C) Tafel curve (D) Electrochemical impedance spectroscopy (E) Electrochemical C-V curve (F) Electrochemical active surface area.

**
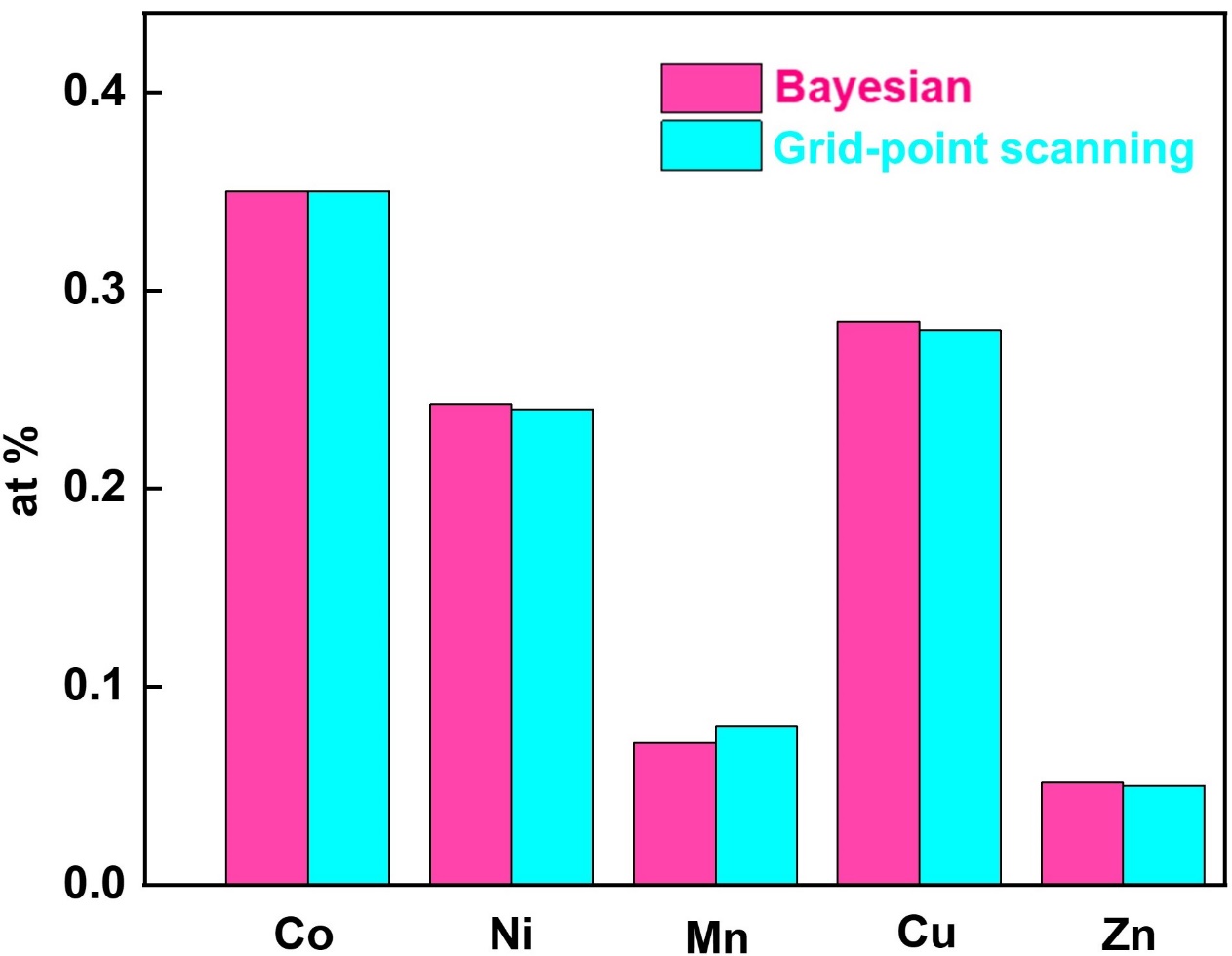
**

**Figure S45.** The comparation of composition ratios between optimal samples suggested by Bayesian optimization and by grid-point scanning. Bayesian optimization is more efficient since it can narrow down the huge searching space rapidly. Moreover, Bayesian optimization can reach to higher resolution than exhaustive grid-point scanning method.


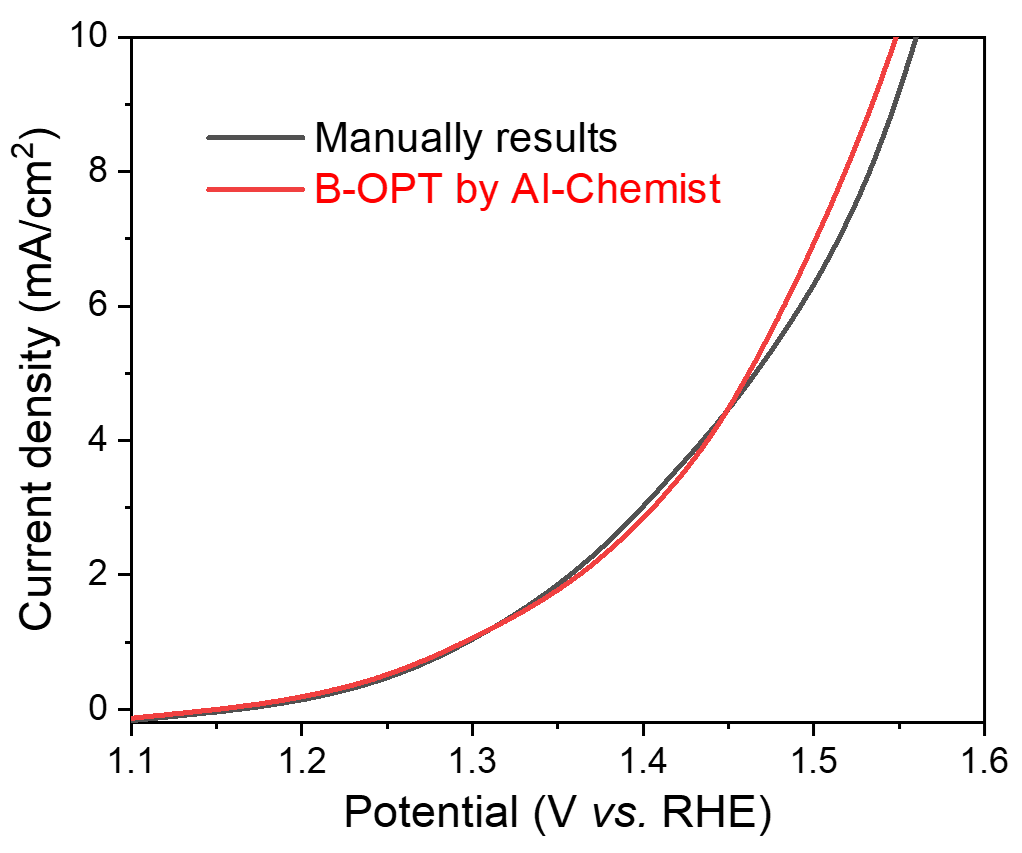


Figure S46. Comparison chart of manual experiment results with the AI-Chemist.


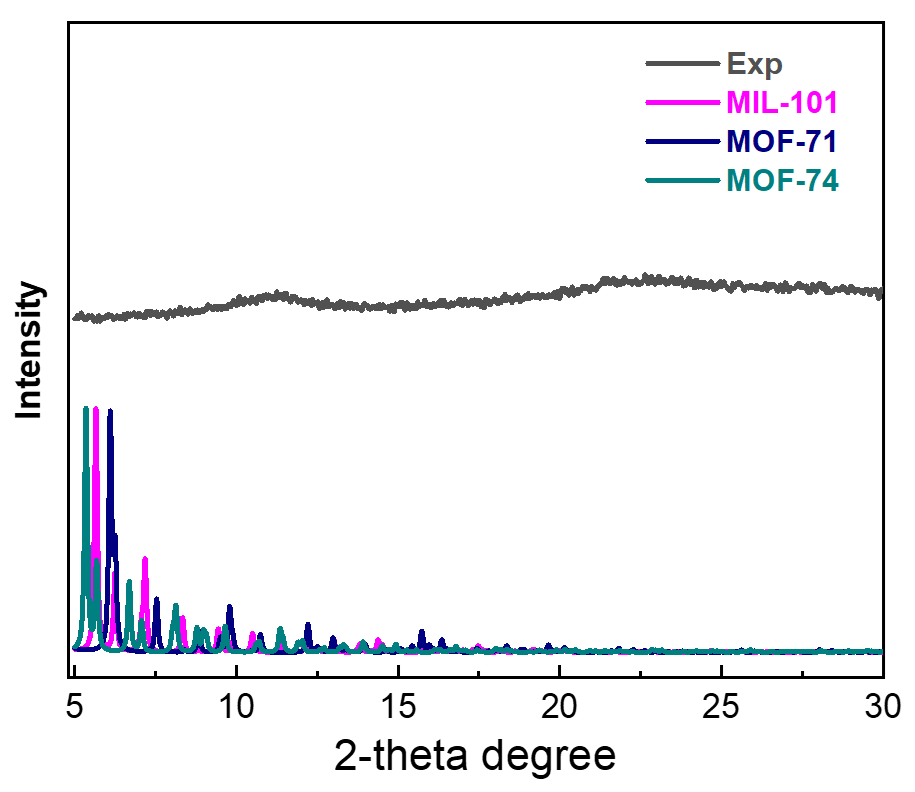


Figure S47. The XRD pattern of B-OPT catalyst and the diffraction peaks beneath are the simulated results from structures of MIL-101, MOF-74 and MOF-71.


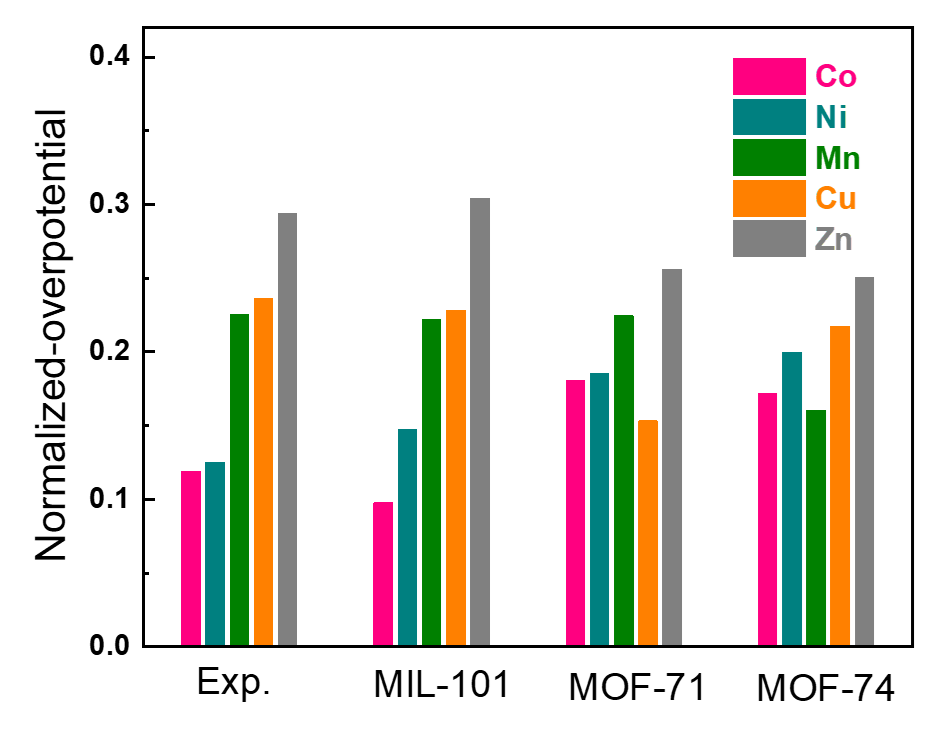


Figure S48. The comparations of normalized overpotential between experiments and simulation results from MIL-101, MOF-71 and MOF-74.

**Table S1.** The comparison chart of characteristics of AI-Chemist with the existing reported automatic chemical experiment system.

|  | Ref[5] | Ref[6] | Ref[7] | Ref[8] | Ref[9] | Ref[10] | Our work |
| --- | --- | --- | --- | --- | --- | --- | --- |
| Experimental  generality | Photocatalysis | Thin-film materials | Organic photovoltaic material | Photocatalysis | Au nanocrystals | Organic  synthesis | Procedure design, synthesis, characterization, testing, and automatic generation reports |
| Experiment plan/process design | null | null | null | null | null | Yes | Yes |
| Multitasking parallelism | null | null | null | null | null | null | Yes |
| Automated synthesis | null | Yes | null | null | Yes | Yes | Yes |
| Robotic arm | Yes | Yes | Yes | null | null | null | Yes |
| Mobile robotics | Yes | null | null | null | null | null | Yes |
| Workstations number | 5 | Integrated sites | Integrated sites | 4 | Integrated sites | Integrated sites | 15 |
| Automated data collection | GC | Optical photography, UV, conductivity, XRF | Optical photograph, UV, J-V curve | GC | UV | chromatographic separation | OER, UV, PL,GC, Raman, and visualization of data graphs |
| Automated data processing | Yes | Yes | Yes | null | null | null | Yes |
| Big database | null | null | null | null | null | Yes | Yes |
| Back-office management | null | null | null | null | null | null | Yes |
| Liquid dispensing error | ±50μl | ±5μl | ±5μl | null | null | null | ±3μl |
| Solid dispensing error | ±0.1mg | null | null | null | null | null | ±0.1mg |
| Intelligent brain and automatic optimization algorithms | Bayesian | Bayesian | Gaussian process regression | null | Genetic Algorithm | null | Neural networks trained on theoretical computational data + Bayesian |

**Table S2.** The top 15 molecules with high occurrence frequencies in the AIE experiment (See Supplementary Data 1 for complete list).


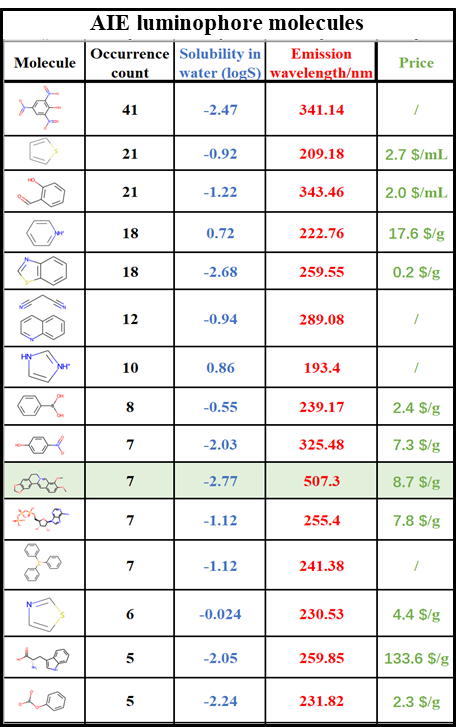


**Table S3.** Experimental data statistics analysis for the photocatalytic H_2_ production and RhB degradation.

| Exp. group | photocatalytic H_2_ production rate (μmol/h) | | | | | Mean Value | Standard Deviation Error | Deviation Percentage |
| --- | --- | --- | --- | --- | --- | --- | --- | --- |
|  | Trial I | Trial II | Trial III | Trial IV | Trial V |  |  |  |
| 1 | 1.85 | 2.01 | 1.79 | 2.04 | 1.88 | 1.914 | 0.10691 | 5.6% |
| 2 | 3.61 | 3.89 | 3.88 | 4.16 | 3.89 | 3.886 | 0.19450 | 5.0% |
| 3 | 6.02 | 5.34 | 5.35 | 5.27 | 5.64 | 5.524 | 0.31150 | 5.6% |
| 4 | 6.86 | 6.58 | 6.54 | 7.12 | 7.45 | 6.910 | 0.38210 | 5.5% |
| 5 | 8.68 | 7.49 | 8.39 | 8.19 | 8.46 | 8.242 | 0.45538 | 5.5% |
| 6 | 10.58 | 10.69 | 9.35 | 9.96 | 10.35 | 10.186 | 0.54455 | 5.3% |
| 7 | 12.56 | 12.41 | 11.21 | 12.45 | 11.98 | 12.122 | 0.55549 | 4.7% |
| 8 | 11.02 | 12.59 | 11.78 | 10.54 | 11.12 | 11.410 | 0.79410 | 7.0% |
| **Average Deviation** | | | | | | | | **5.5%** |
|  | | | | | | | | |
| Exp. group | photocatalytic degradation efficiency of RhB (%) | | | | | Mean Value | Standard Deviation Error | Deviation Percentage |
|  | Trial I | Trial II | Trial III | Trial IV | Trial V |  |  |  |
| 1 | 48.36 | 55.61 | 55.23 | 49.36 | 46.11 | 50.934 | 4.26305 | 8.4% |
| 2 | 77.96 | 75.39 | 72.16 | 68.21 | 73.64 | 73.472 | 3.64894 | 5.0% |
| 3 | 61.35 | 56.34 | 55.42 | 66.32 | 58.67 | 59.620 | 4.39272 | 7.4% |
| 4 | 46.37 | 38.69 | 42.16 | 44.63 | 39.36 | 42.242 | 3.30425 | 7.8% |
| 5 | 29.56 | 32.55 | 31.29 | 29.28 | 25.67 | 29.670 | 2.60207 | 8.8% |
| 6 | 11.35 | 9.89 | 11.23 | 11.39 | 13.03 | 11.378 | 1.11424 | 9.8% |
| 7 | 14.52 | 14.32 | 15.14 | 16.13 | 12.45 | 14.512 | 1.35103 | 9.3% |
| 8 | 14.98 | 13.89 | 17.55 | 15.89 | 17.27 | 15.916 | 1.53986 | 9.7% |
| **Average Deviation** | | | | | | | | **8.3%** |

**Table S4.** DFT calculations of three catalytic properties of cluster MOFs of three metals, the three catalytic properties represent the free energy change of hydroxyl adsorption, the difference between G_O*_ and G_OH*_ and the charge transfer during hydroxyl adsorption the activate site metal, respectively.

| **Metal** | **G_OH*_ / eV** | **G_O*-OH*_ / eV** | **Δe / e** |
| --- | --- | --- | --- |
| Co-Co-Co | 1.92726 | 1.55073 | 0.03848 |
| Co-Co-Ni | 2.08770 | 1.57548 | 0.02051 |
| Co-Co-Mn | 1.90587 | 1.55604 | 0.07838 |
| Co-Co-Cu | 2.10493 | 1.91646 | 0.00698 |
| Co-Co-Zn | 2.25957 | 1.61561 | 0.16752 |
| Ni-Co-Ni | 1.89802 | 1.83905 | 0.05378 |
| Ni-Co-Mn | 1.99932 | 1.76248 | 0.05096 |
| Ni-Co-Cu | 1.97925 | 1.97944 | -0.01568 |
| Ni-Co-Zn | 1.96170 | 1.92653 | 0.06633 |
| Mn-Co-Mn | 2.85557 | 2.16488 | 0.08423 |
| Mn-Co-Cu | 2.03251 | 2.05799 | -0.15302 |
| Mn-Co-Zn | 1.32881 | 1.70676 | 0.05910 |
| Cu-Co-Cu | 2.16893 | 1.90477 | -0.08985 |
| Cu-Co-Zn | 2.15493 | 1.92637 | 0.02123 |
| Zn-Co-Zn | 2.42413 | 1.90308 | -0.01495 |
| Ni-Ni-Ni | 2.20230 | 2.28318 | 0.08789 |
| Ni-Ni-Mn | 2.53418 | 1.90762 | 0.04766 |
| Ni-Ni-Cu | 2.33274 | 2.11223 | 0.06806 |
| Ni-Ni-Zn | 2.33678 | 2.11627 | 0.06677 |
| Mn-Ni-Mn | 2.21438 | 2.20121 | 0.07402 |
| Mn-Ni-Cu | 2.15812 | 2.04605 | 0.07821 |
| Mn-Ni-Zn | 2.38192 | 2.18916 | 0.01563 |
| Cu-Ni-Cu | 2.51273 | 2.06814 | 0.06644 |
| Cu-Ni-Zn | 2.38304 | 2.31357 | 0.09150 |
| Zn-Ni-Zn | 2.36906 | 2.22144 | 0.07802 |
| Mn-Mn-Mn | 2.81582 | 0.17720 | 0.01556 |
| Mn-Mn-Cu | 2.10434 | 2.14460 | 0.07943 |
| Mn-Mn-Zn | 2.02568 | 2.25657 | 0.05694 |
| Cu-Mn-Cu | 2.11640 | 2.11864 | 0.05490 |
| Cu-Mn-Zn | 2.31781 | 2.08957 | 0.08389 |
| Zn-Mn-Zn | 2.43179 | 2.13618 | 0.00909 |
| Cu-Cu-Cu | 2.85902 | 2.16079 | 0.08555 |
| Cu-Cu-Zn | 2.90773 | 2.19139 | 0.07631 |
| Zn-Cu-Zn | 2.81952 | 2.28081 | -0.01596 |
| Zn-Zn-Zn | 3.40220 | 1.99641 | 0.04148 |

# References

1. University of Science and Technology of China., Automatic Control Software for Electrochemical Workstation. CN Patent 2021SR2139156, issued Dec. 24, 2021.

2. Garrido-Jurado, S, Muñoz-Salinas, R, Madrid-Cuevas, FJ*, et al.* Automatic generation and detection of highly reliable fiducial markers under occlusion. *Pattern Recogn*. 2014; **47**(6): 2280-92.

3. Sajjan, S, Moore, M, Pan, M*, et al.* Clear grasp: 3d shape estimation of transparent objects for manipulation. In: *2020 IEEE International Conference on Robotics and Automation (ICRA),* *2020*, p. 3634-42. IEEE.

4. Liu, X, Jonschkowski, R, Angelova, A*, et al.* Keypose: Multi-view 3d labeling and keypoint estimation for transparent objects. In: *Proceedings of the IEEE/CVF conference on computer vision and pattern recognition,* *2020*, p. 11602-10.

5. Burger, B, Maffettone, PM, Gusev, VV*, et al.* A mobile robotic chemist. *Nature*. 2020; **583**(7815): 237-241.

6. MacLeod, BP, Parlane, FGL, Morrissey, TD*, et al.* Self-driving laboratory for accelerated discovery of thin-film materials. *Sci Adv*. 2020; **6**(20).

7. Du, XY, Luer, L, Heumueller, T*, et al.* Elucidating the Full Potential of OPV Materials Utilizing a High-Throughput Robot-Based Platform and Machine Learning. *Joule*. 2021; **5**(2): 495-506.

8. Sahm, CD, Ucoski, GM, Roy, S*, et al.* Automated and Continuous-Flow Platform to Analyze Semiconductor-Metal Complex Hybrid Systems for Photocatalytic CO_2_ Reduction. *Acs Catal*. 2021; **11**(17): 11266-77.

9. Salley, D, Keenan, G, Grizou, J*, et al.* A nanomaterials discovery robot for the Darwinian evolution of shape programmable gold nanoparticles. *Nat Commun*. 2020; **11**(1).

10. Rohrbach, S, Šiaučiulis, M, Chisholm, G*, et al.* Digitization and validation of a chemical synthesis literature database in the ChemPU. *Science*. 2022; **377**(6602): 172-80.
